# Culture and identification of *Borrelia *spirochetes in human vaginal and seminal secretions

**DOI:** 10.12688/f1000research.5778.3

**Published:** 2015-04-27

**Authors:** Marianne J. Middelveen, Jennie Burke, Eva Sapi, Cheryl Bandoski, Katherine R. Filush, Yean Wang, Agustin Franco, Arun Timmaraju, Hilary A. Schlinger, Peter J. Mayne, Raphael B. Stricker

**Affiliations:** 1 International Lyme and Associated Diseases Society, Bethesda, MD, 20827-1461, USA; 2 Australian Biologics, Sydney, NSW 2000, Australia; 3 Department of Biology and Environmental Science, University of New Haven, West Haven, CT, 06516, USA

**Keywords:** Lyme borreliosis, chronic Lyme disease, Borrelia burgdorferi, spirochetes, sexual transmission.

## Abstract

**Background: **Recent reports indicate that more than 300,000 cases of Lyme disease are diagnosed yearly in the USA. Preliminary clinical, epidemiological and immunological studies suggest that infection with the Lyme disease spirochete *Borrelia burgdorferi* (Bb) could be transferred from person to person via intimate human contact without a tick vector. Failure to detect viable *Borrelia* spirochetes in vaginal and seminal secretions would argue against this hypothesis.

**Methods: **Patients with and without a history of Lyme disease were selected for the study after informed consent was obtained. Serological testing for Bb was performed on all subjects. Semen or vaginal secretions were inoculated into BSK-H medium and cultured for four weeks. Examination of genital cultures and culture concentrates for the presence of spirochetes was performed using light and darkfield microscopy, and spirochete concentrates were subjected to Dieterle silver staining, anti-Bb immunohistochemical staining, molecular hybridization and PCR analysis for further characterization. Immunohistochemical and molecular testing was performed in three independent laboratories in a blinded fashion. Positive and negative controls were included in all experiments.

**Results: **Control subjects who were asymptomatic and seronegative for Bb had no detectable spirochetes in genital secretions by PCR analysis. In contrast, spirochetes were observed in cultures of genital secretions from 11 of 13 subjects diagnosed with Lyme disease, and motile spirochetes were detected in genital culture concentrates from 12 of 13 Lyme disease patients using light and darkfield microscopy. Morphological features of spirochetes were confirmed by Dieterle silver staining and immunohistochemical staining of culture concentrates. Molecular hybridization and PCR testing confirmed that the spirochetes isolated from semen and vaginal secretions were strains of *Borrelia*, and all cultures were negative for treponemal spirochetes. PCR sequencing of cultured spirochetes from three couples having unprotected sex indicated that two couples had identical strains of Bb *sensu stricto* in their semen and vaginal secretions, while the third couple had identical strains of *B. hermsii* detected in their genital secretions.

**Conclusions: **The culture of viable *Borrelia* spirochetes in genital secretions suggests that Lyme disease could be transmitted by intimate contact from person to person. Further studies are needed to evaluate this hypothesis.

## Introduction

Lyme disease is the most common human tick-borne disease in the world today ( Stricker & Johnson, 2014). It is transmitted by *Ixodes* ticks and is caused by the spirochete *Borrelia burgdorferi* (Bb) ( Burgdorfer *et al.*, 1982). Bb is phylogenetically related to the spirochetal agent of syphilis, *Treponema pallidum* ( Gupta *et al.*, 2013). *T. pallidum* is transmitted sexually between partners through contact of mucosal membranes, gaining access to the bloodstream through microabrasions and then disseminating systemically ( Ho & Lukehart, 2011; LaFond & Lukehart, 2006). The close phylogenic relationship of Bb to *T. pallidum* suggests that this mode of transmission might be possible for Bb.

In addition to theoretical considerations, evidence for non-vector transmission of Bb is based on animal models. Proof of contact transmission of Bb – without involvement of an arthropod vector – was established by two studies in mice. Burgess *et al.* (1986) caged uninfected deer mice with experimentally-infected deer mice and demonstrated transmission of Bb by seroconversion of contact-exposed mice from negative to positive and by the isolation of Bb from the blood of one contact-exposed mouse 42 days after initial contact. A study by Wright & Nielsen (1990) demonstrated that white-footed mice were susceptible to oral infection and transmitted infection to each other through direct contact. Furthermore, sexual transmission of Bb has been proposed in a canine model. Bb was transmitted to uninfected female dogs in estrus via semen by natural breeding with male dogs infected experimentally with Bb ( Gustafson, 1993). Successful transmission of infection from male dogs to female dogs was shown by seroconversion of female dogs from negative to positive as well as the detection of Bb DNA in the tissue of fetuses from resulting pregnancies. If contact transmission of Bb occurs in mice and sexual transfer occurs in dogs, it is not unreasonable to postulate similar routes of infection in humans.

We sought to determine if viable *Borrelia* spirochetes could be recovered from human vaginal and seminal secretions, an important first step to investigate whether sexual transmission of these spirochetes among humans is possible.

## Materials and methods

### 1. Research subject selection

Control subjects who were asymptomatic without a history of Lyme disease and patients with a history of Lyme disease were recruited for the study after written informed consent to collect and publish their data was obtained. Approval for sample collection was obtained from the Western Institutional Review Board, Olympia, WA (WIRB ^®^ #20141439). Further approval for sample testing was obtained from the Institutional Review Board of the University of New Haven, West Haven, CT. Serological testing of all participants after coding of their blood samples was performed by IGeneX Reference Laboratories, Palo Alto, CA in a blinded fashion.

Patients were considered positive for Lyme disease if they were serologically positive by CDC criteria and/or IGeneX criteria, as previously described ( Engstrom *et al.*, 1995; Ma *et al.*, 1992), or if they had musculoskeletal, neurocognitive and/or cardiac symptoms clinically consistent with a Lyme disease diagnosis, as described elsewhere ( Donta, 2014; Smith *et al.*, 2014). None of the patients were taking antibiotics at the time of testing.

### 2. *Borrelia* cultures

*Borrelia* spirochetes were cultured as previously described ( Bankhead & Chaconas, 2007; Middelveen *et al.*, 2013b; Middelveen *et al.*, 2014a). The inoculum for blood culture was prepared as follows: 10 milliliters of whole blood was collected by sterile venipuncture from each patient. Samples sat at room temperature for 10 to 15 minutes allowing clotting to occur. Red blood cells (RBCs) were separated by low speed centrifugation. Barbour–Stoner–Kelly H (BSK-H) complete medium was used for cultures with the addition of 6% rabbit serum (Sigma Aldrich, #B8291) and the following antibiotics: phosphomycin (0.02 mg/ml), rifampicin (0.05 mg/ml), and amphotericin B (2.5 µg/ml) (Sigma Aldrich).

The culture medium described above was inoculated for blood culture with the spun serum containing white blood cells and some RBCs, and for genital culture with either ejaculated semen or vaginal secretions collected by intravaginal swabbing with a sterile cotton-tipped swab. Blood and genital cultures were incubated at 32°C in an Oxoid anaerobic jar (Thermo Scientific) containing an AnaeroGen sachet (Thermo Scientific) to provide an anaerobic environment. Cultures were incubated for four weeks and checked weekly by light and/or darkfield microscopy for visible motile spirochetes.

All cultures were processed for microscopic imaging and PCR by centrifuging the culture fluid at 15,000 g for 20 minutes to concentrate spirochetes. The supernatant was discarded and the pellet retained. The pellet samples were coded and processed in a blinded fashion for subsequent experiments.

### 3. Dieterle silver staining

Dieterle silver staining was performed using two fixation methods. In the standard method, formalin-fixed, paraffin-embedded pellets were sectioned and stained with Dieterle silver stain as previously described ( Aberer & Duray, 1991; Middelveen *et al.*, 2013a). In the newer method, culture fluid was spread and dried on a SuperFrost™ Plus microscope slide (Fisher Scientific) and fixed by incubating the slide in acetone for 10 minutes at -20°C, as previously described ( Sapi *et al.*, 2013). Dieterle silver staining was performed on the acetone-fixed slide.

Positive and negative culture controls were prepared for comparison purposes with plasma from Bb-inoculated mice and uninfected mice followed by Dieterle silver staining using the standard method. Control cultures of mixed Gram-positive and mixed Gram-negative bacteria were also subjected to Dieterle staining. The control processing and staining was performed at McClain Laboratories LLC, Smithtown, NY.

### 4. Anti-Bb immunostaining

***A. McClain Laboratories.*** Blood and genital culture pellets from coded patient samples were processed in a blinded fashion for special staining at McClain Laboratories. Formalin-fixed, paraffin-embedded pellets were sectioned and stained with anti-Bb immunostain for spirochete detection, as previously described ( Middelveen *et al.*, 2013a; Middelveen *et al.*, 2014a). In brief, immunostaining was performed using an unconjugated rabbit anti-Bb polyclonal antibody (Abcam ab20950), incubated with an alkaline phosphatase probe (Biocare Medical #UP536L), followed by a chromogen substrate (Biocare Medical #FR805CHC), and counterstained with hematoxylin. Positive and negative culture controls were prepared for comparison purposes with plasma from Bb-inoculated mice and uninfected mice followed by anti-Bb immunostaining. Culture pellets from fungal-infected human skin samples, mixed Gram-positive bacteria and mixed Gram-negative bacteria were also prepared for comparison purposes as negative anti-Bb immunostain controls to exclude cross-reactivity with commonly encountered microorganisms. Staining was titrated to determine optimal antibody dilutions to achieve positive staining of spirochetes while minimizing background staining ( Middelveen *et al.*, 2013a; Middelveen *et al.*, 2014a).

***B. University of New Haven.*** Coded samples were processed in a blinded fashion for Bb immunostaining as previously described ( Sapi *et al.*, 2013). Culture fluid was spread and dried on a SuperFrost™ Plus microscope slide (Fisher Scientific) and fixed by incubating the slide in acetone for 10 minutes at -20°C. Dried, fixed culture fluid was submerged under 100 μl of polyclonal FITC-labeled rabbit anti-Bb antibody (Thermo Scientific #PA-1-73005) diluted 1:50 in 1× PBS buffer with 1% BSA (Sigma Aldrich #A9418). For negative controls, the antibody was omitted and replaced with normal rabbit serum. The slides were then incubated for 1 hour at 37°C in a humidified chamber, washed with 1× PBS for 5 minutes at room temperature, rinsed twice in double distilled water and dried in a laminar air-flow hood for 10 minutes. The slides were mounted with Vectashield mounting medium (Vector Labs) and viewed with fluorescent microscopy at 400× magnification with a Leica DM2500 microscope ( Sapi *et al.*, 2013).

### 5. Molecular hybridization using Bb DNA probe

The Bb molecular beacon DNA probe was generously provided by Dr. Alan MacDonald. Probe FlaB (sequence of 23 mer TGGGAGTTTCTGGTAAGATTAAT) was derived from the Bb open reading frame (ORF) BB0147 (approximately 1100 mer) of the flagellin B gene. A nucleotide Basic Local Alignment Search Tool (BLAST) search of the 23 mer sequence disclosed no matches in the human genome or in any other life form other than the Bb sequence of BB0147.

Bb detection with the molecular beacon was performed as previously described ( Middelveen *et al.*, 2014a) on coded samples in a blinded fashion using the following protocol: paraffin sections were dewaxed by baking at 60°C, then immersed in serial 100% xylene baths followed by serial immersion through baths of 100% ethanol, 90% ethanol, 80% ethanol, and finally in distilled H _2_O, and then air-dried. Fixed sections were immersed in 20 μl of the working DNA beacon solution. The sectioned specimen was covered with a layer of plastic cut from a Ziploc ^®^ freezer bag and was heated at 90°C for 10 minutes to denature DNA and RNA. The heat was first reduced to 80°C for 10 minutes, then the slides were removed from heat and allowed to gradually cool to 24°C. The slides were washed in PBS, covered with 30% glycerol and a glass coverslip, then examined under an EPI Fluor microscope. Staining of test specimens was performed alongside staining of positive and negative controls. The positive control was prepared by embedding a known Bb strain in agarose, formalin-fixing the specimen then blocking in paraffin and staining sections as described above.

**Supplemental Figure 1.**
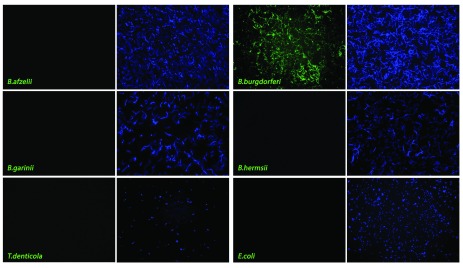
Molecular hybridization of FlaB probe with *Borrelia* strains, *T. denticola* and *E.coli*. FlaB hybridization is shown in green, while DAPI counterstain of bacterial targets is shown in blue. Note specific hybridization of FlaB probe with *B. burgdorferi sensu stricto* and lack of hybridization with other *Borrelia* strains, *T. denticola* or *E.coli*. 400× magnification. See Dataset, data file 4.

The specificity of the FlaB probe was validated in studies performed at the University of New Haven (Sapi E., unpublished observation 2014; see Supplemental Figure 1). The FlaB probe hybridized to Bb *sensu stricto*, yet failed to hybridize with *B. afzelii*, *B. garinii*, *B. hermsii*, *Treponema denticola* and *Escherichia coli*. Thus the probe appears to be specific for detection of Bb *sensu stricto*.

### 6. PCR of cultures

Blood and genital culture pellets were first dissolved in 200 μl of Qiagen buffer, then forwarded to the University of New Haven, Department of Biology and Environmental Science, West Haven, CT, USA and Australian Biologics, Sydney, NSW, Australia for PCR detection of *Borrelia*. All control and patient samples were coded, and PCR testing was performed in a blinded fashion.

***A. Australian Biologics.*** Detection of *Borrelia* by PCR was performed as previously described ( Mayne *et al.*, 2012) using the Eco™ Real-Time PCR system with primers targeted to the genes encoding 16S rRNA ( *Borrelia*), flA ( *T. denticola*) and fliG1 ( *T. pallidum*) and analyzed with the software version 3.0.16.0. DNA was extracted from the dissolved culture pellets using the QIAamp DNA Mini Kit (Qiagen) and 20 μl were used for each reaction. The thermal profile involved incubation for 2 minutes at 50°C, polymerase activation for 10 minutes at 95°C then PCR cycling for 40 cycles of 10 seconds at 95°C dropping to 60°C sustained for 45 seconds. All samples were run in duplicate with positive and negative controls. Positive controls were genomic DNA samples from *B. burgdorferi, B. garinii, and B. afzelii* (Amplirun DNA/RNA amplification controls, Vircell S.L, Granada, Spain). Negative controls were samples of non-template DNA in molecular-grade water. The magnitude of the PCR signal generated (∆R) for each sample was interpreted as positive or negative compared to positive and negative controls.

In samples with sufficient DNA for sequencing, endpoint PCR amplification and Sanger sequencing of the *Borrelia* gene target from cultures was followed by BLAST comparison with known *Borrelia* sequences, as previously described ( Mayne *et al.*, 2012).

***B. University of New Haven.*** DNA samples were extracted from blood, vaginal or seminal cultures by lysing cells overnight in 180 µl tissue lysis buffer (Qiagen) and 20 µl Proteinase K (Qiagen) at 56°C in a shaking water bath followed by phenol:chloroform extraction the next day. The DNA was resuspended in 50–100 µl 1×TE buffer.

A published TaqMan assay targeting a 139-bp fragment of the gene encoding the *Borrelia* 16S rRNA was used for the detection of *Borrelia* in DNA extracted from patient samples ( O’Rourke *et al.*, 2013). All reactions were carried out at a final volume of 20 µl and consisted of 900 nM of each primer, 200 nM of probe, and 10 µl of 2× TaqMan Universal PCR Master Mix (Applied Biosystems) and 1 nanogram of DNA. Amplifications were carried out on a CFX96 Real-Time System (Bio-Rad), and cycling conditions consisted of 50°C for 2 minutes, 95°C for 10 minutes, followed by 40 cycles of 95°C for 15 seconds and 60°C for 60 seconds. Fluorescent signals were recorded with CFX96 Real-Time software and Cq threshold was set automatically. The reactions were performed in triplicate with positive and negative controls.

Nested PCR primers for the genes encoding the *Borrelia* 16S rRNA, *fla* and *pyrG* loci were used as previously described ( Clark *et al.*, 2013; Margos *et al.*, 2010; Sapi *et al.*, 2013). Reactions were carried out in a final volume of 50 µl using 10 µl template DNA. Final concentrations were 2× Buffer B (Promega), 2 mM MgCl _2_, 0.4 mM dNTP mix, 2 µM of each primer, and 2.5 U Taq polymerase (Invitrogen). “Outer” primers were used in the first reaction. “Inner” primers were used for the nested reaction, in which 1 µl of PCR product from the first reaction was used as template for the second. Cycling parameters were as follows: 94°C for 5 minutes followed by 40 cycles of denaturation at 94°C for 1 minute, annealing for 1 minute (temperature based on the primer set used), and extension at 72°C for 1 minute, with a final extension step at 72°C for 5 minutes. PCR products were visualized on 1–2% agarose gels. Sanger sequencing was used for gene analysis, as previously described ( Margos *et al.*, 2010).

## Results

### 1. Patient data

All patient data are shown in Table 1. The control group included four asymptomatic patients (two males and two females). All four were seronegative for Bb.

**Table 1.** Patient Data. Patients 6 & 7 (*), 8 & 9 (**), 10 & 11 (†), and 12 & 13 (††) are sexual partners. Patients 8 and 11 were seronegative but clinically diagnosed with Lyme disease.

Control	Sex	Age	Serology
1 (M)	male	63	negative
2 (M)	male	53	negative
3 (F)	female	58	negative
4 (F)	female	43	negative

**Patient**	**Sex**	**Age**	**Serology**
1 (F)	female	56	equivocal
2 (M)	male	45	positive
3 (M)	male	35	positive
4 (F)	female	66	positive
5 (F)	female	27	positive
6 (M)*	male	63	positive
7 (F)*	female	53	positive
8 (M)**	male	42	negative
9 (F)**	female	40	positive
10 (M)†	male	56	positive
11 (F)†	female	54	negative
12 (M)††	male	65	positive
13 (F)††	female	54	positive

The patient group included six male subjects and seven female subjects, including four pairs of partners (Patients 6 and 7, 8 and 9, 10 and 11, and 12 and 13, respectively). Eleven of the 13 patients selected for the study were serologically positive for Lyme disease. Patient 1 was serologically equivocal and patient 8 was seronegative, although Bb plasmid DNA was detected in whole blood and serum from this patient.

### 2. Light and darkfield microscopy

**Figure 1.**
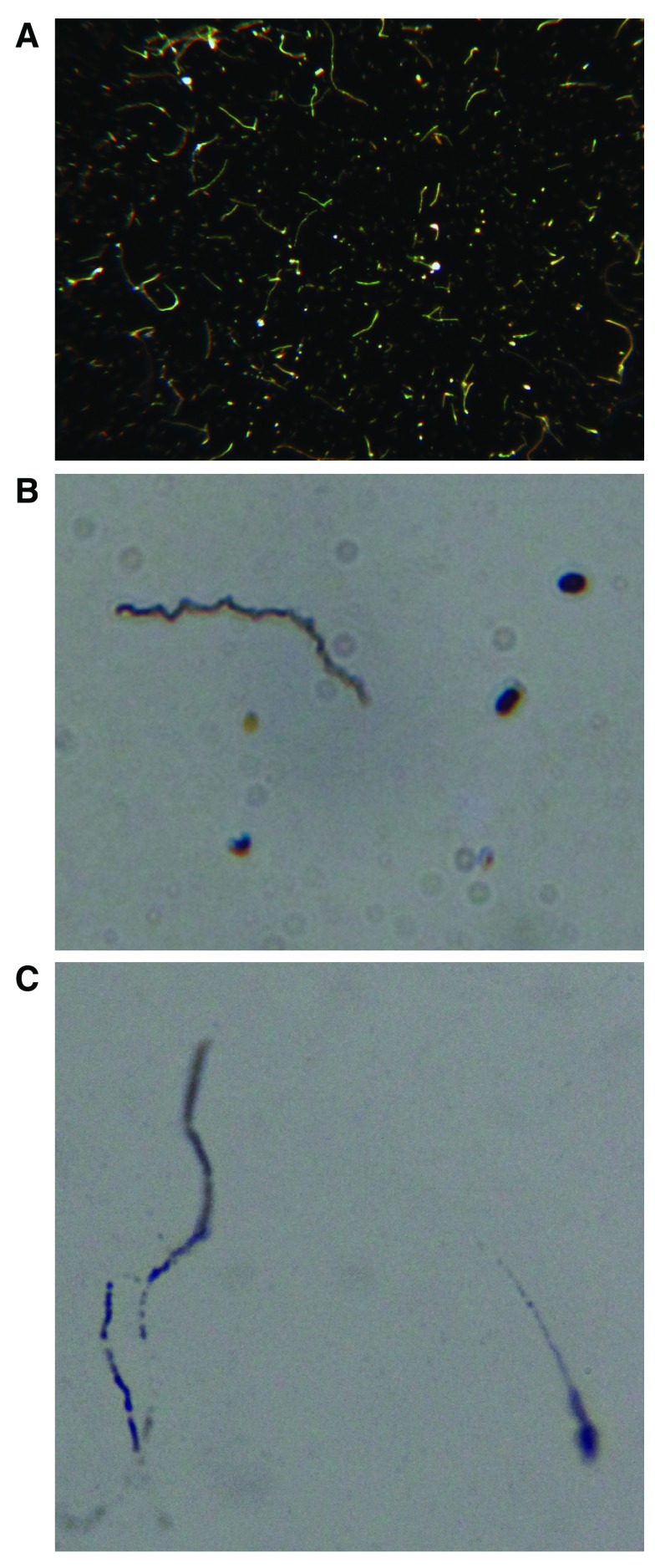
**A**: Darkfield image of genital culture from Patient 1. Note numerous spirochetes. 400× magnification. See Dataset, data file 1. **B**: Dieterle silver stain of genital culture from Patient 12. Note darkly staining spirochete. Formalin fixed slide, 400× magnification. See Dataset, data file 2. **C**: Semen sample from Patient 10 showing *B. burgdorferi* spirochetes (left) and sperm cell (right). Dieterle silver stain of acetone fixed slide, 1000× magnification. See Dataset, data file 2.

Blood cultures from 11 patients were incubated for four weeks and checked weekly for spirochete growth using light and darkfield microscopy. Motile spirochetes and/or motile spherules were observed in the culture fluid from all 11 patients after four weeks ( Table 2). Genital cultures from the four controls were incubated for four weeks. None of the control cultures contained visible spirochetes, and the cultures were sent for PCR testing. Genital cultures from the 11 patients were incubated for four weeks and checked weekly. Motile spirochetes were observed in the culture fluid from all 11 patients after four weeks ( Figure 1A). See Dataset, data file 1.

**Table 2.** Microscopy results from fresh blood and genital culture fluid. See Dataset, data file 1. ND, not done.

Patient number	Microscopy – fresh blood culture fluid	Microscopy – fresh genital culture fluid
1 (F)	motile spherules	vaginal – motile spirochetes
2 (M)	ND	seminal – ND
3 (M)	ND	seminal – ND
4 (F)	motile spherules	vaginal – motile spherules and spirochetes
5 (F)	motile spherules	vaginal – motile spirochetes, some yeast cells
6 (M)	motile spirochetes and spherules	seminal – motile spirochetes
7 (F)	motile spirochetes and spherules	vaginal – motile spherules and spirochetes
8 (M)	motile spherules	seminal – motile spirochetes
9 (F)	motile spherules	vaginal – motile spirochetes
10 (M)	motile spherules	seminal – motile spherules/ spirochetes
11 (F)	motile spherules	vaginal – motile spherules/ spirochetes
12 (M)	motile spherules	seminal – motile spherules/ spirochetes
13 (F)	motile spherules	vaginal – motile spherules/ spirochetes

Most genital cultures grew very well and contained abundant spirochetes, but some blood cultures contained few spirochetes. Therefore, to better document the presence of spirochetes in culture, the culture fluid was concentrated into pellets by centrifugation ( Table 3). Spirochetes and/or spherules were detected by sectioning and special staining of paraffin blocked pellets in all the patient blood and genital cultures concentrated by centrifugation, except for blood and genital culture pellets from Patient 1 that were lost during paraffin blocking ( Table 3). Control genital culture samples were sent directly for PCR testing and were not subjected to light and darkfield microscopy.

**Table 3.** Microscopy results and Dieterle silver staining of genital culture concentrates. See Dataset, data file 2.

Patient number	Microscopy – genital culture pellet	Dieterle silver stain – genital culture pellet
1 (F)	pellet lost	pellet lost
2 (M)	seminal – spherules/ spirochetes	seminal – spherules/ spirochetes
3 (M)	seminal – spirochetes	seminal – spherules/ spirochetes
4 (F)	vaginal – spirochetes	vaginal – spherules
5 (F)	vaginal – spirochetes, some yeast cells	vaginal – spherules
6 (M)	seminal – spirochetes	seminal – spherules/ spirochetes
7 (F)	vaginal – spirochetes	vaginal – spherules/ spirochetes
8 (M)	seminal – spirochetes	seminal – spherules/ spirochetes
9 (F)	vaginal – spherules/ spirochetes	vaginal – spherules
10 (M)	seminal – spirochetes	seminal – spherules/ spirochetes
11 (F)	vaginal – spirochetes	vaginal – spherules/ spirochetes
12 (M)	seminal – spirochetes	seminal – spherules/ spirochetes
13 (F)	vaginal – spirochetes	vaginal – spherules/ spirochetes

### 3. Immunohistochemistry

***A. Dieterle silver staining.*** The culture samples of uninfected mouse plasma, mixed Gram-positive bacteria and mixed Gram-negative bacteria failed to stain with Dieterle silver stain using the standard staining method. In contrast, the culture sample of Bb-infected mouse plasma stained positive for spirochetes with Dieterle silver stain ( Dataset, data file 2A).

Using standard Dieterle staining, spherules and/or spirochetal forms were visible in all patient genital cultures ( Figure 1B). Spirochetes were detected in all patient genital culture pellets except for Patient 1, whose pellet was lost during processing ( Table 3). Using the newer fixation method, spirochetes and sperm cells were visible in semen samples and showed distinct morphology ( Figure 1C). Sperm cells are known to stain with silver stains ( Pathak *et al.*, 1979; Schmid *et al.*, 1983). Sperm cells were seen in all semen samples except for Patients 2 and 6, who had vasectomies (data not shown). Since control genital cultures had no visible spirochetes, the control samples were sent directly for PCR testing and were not subjected to Dieterle silver staining. See Dataset, data file 2.

***B. Anti-Bb immunostaining.***

**I. Culture fluid – University of New Haven**

**Figure 2.**
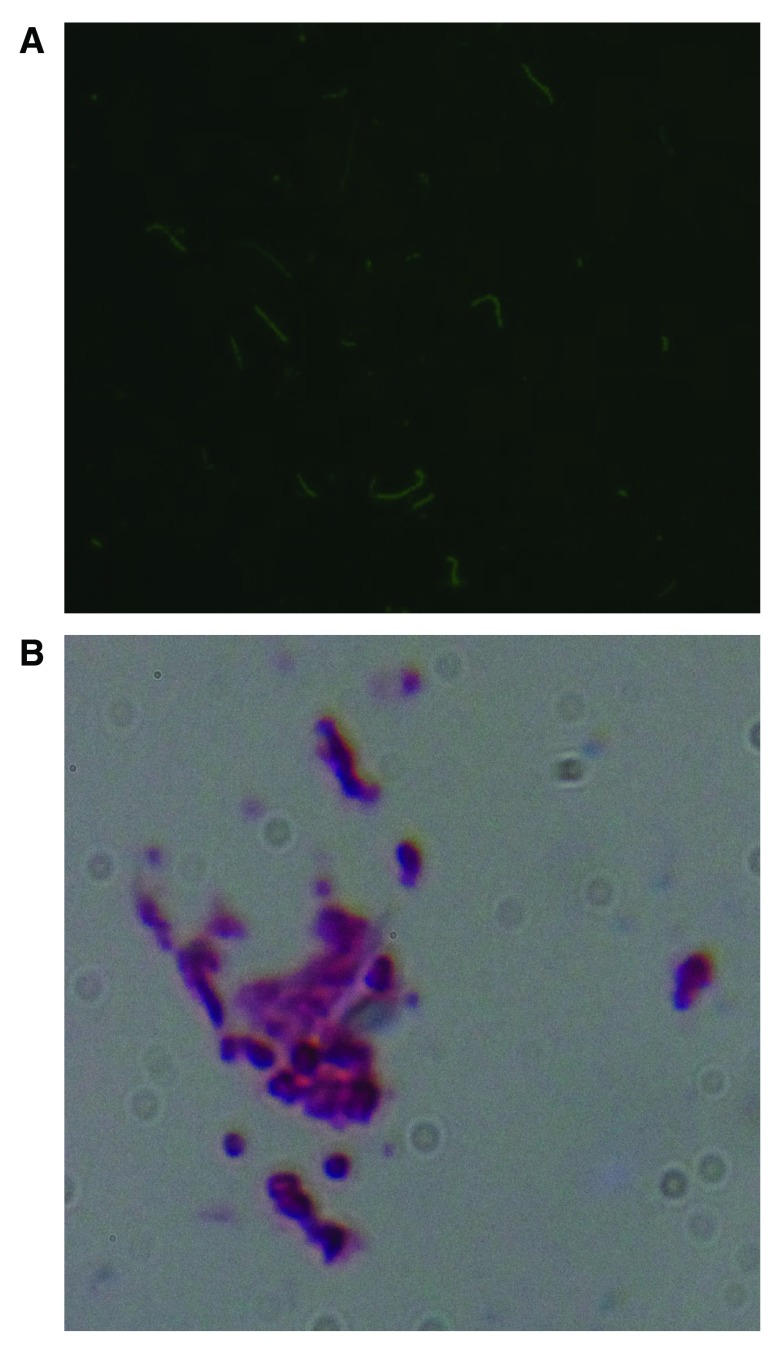
**A**: *B. burgdorferi* immunostaining of vaginal culture from Patient 1. Note intensely staining spiral and round forms in culture. 400× magnification. **B**: *B. burgdorferi* immunostaining of seminal culture from Patient 6. Note intensely staining spiral and round forms in culture. 400× magnification. See Dataset, data file 3.

Genital culture fluid from Patient 1 was fixed on a SuperFrost™ Plus microscope slide and was stained with FITC-labelled polyclonal anti-Bb antibody. Staining was strongly positive, revealing well-defined spirochetes morphologically consistent with Bb ( Figure 2A). The polyclonal antibody was not reactive to *T. denticola* (data not shown).

**II. Culture pellets – McClain laboratories**

The culture sample of uninfected mouse plasma failed to stain with anti-Bb immunostain. In contrast, the culture sample of Bb-infected mouse plasma stained positive for spirochetes with anti-Bb immunostain ( Dataset, data file 3A). Control fungalinfected human skin cultures, Gram-positive bacterial cultures and Gram-negative bacterial cultures all failed to stain for spirochetes with the anti-Bb immunostain ( Dataset, data file 3A).

Anti-Bb immunostaining was positive for all genital cultures except for Patient 1, whose pellet was lost during processing ( Table 4). Immunostaining revealed both spiral and globular Bb forms ( Figure 2B). Since control genital cultures had no visible spirochetes, the control samples were sent directly for PCR testing and were not subjected to immunostaining. See Dataset, data file 3.

**Table 4.** Results of *B. burgdorferi* immunostaining and FlaB molecular hybridization in genital culture concentrates. See Dataset, data files 3 and 4. ND, not done.

Patient number	Bb immunostaining – genital culture pellet	FlaB hybridization – genital culture pellet
1 (F)	pellet lost*	pellet lost
2 (M)	seminal – positive	seminal – positive
3 (M)	seminal – positive	seminal – positive
4 (F)	vaginal – positive	vaginal – positive
5 (F)	vaginal – positive	vaginal – positive
6 (M)	seminal – positive	seminal – positive
7 (F)	vaginal – positive	vaginal – positive
8 (M)	seminal – positive	seminal – positive
9 (F)	vaginal – positive	vaginal – positive
10 (M)	seminal – positive	ND
11 (F)	vaginal – positive	ND
12 (M)	seminal – positive	ND
13 (F)	vaginal – positive	ND

*Positive Bb immunostaining of genital culture fluid. See Results section.

### 4. Molecular hybridization

**Figure 3.**
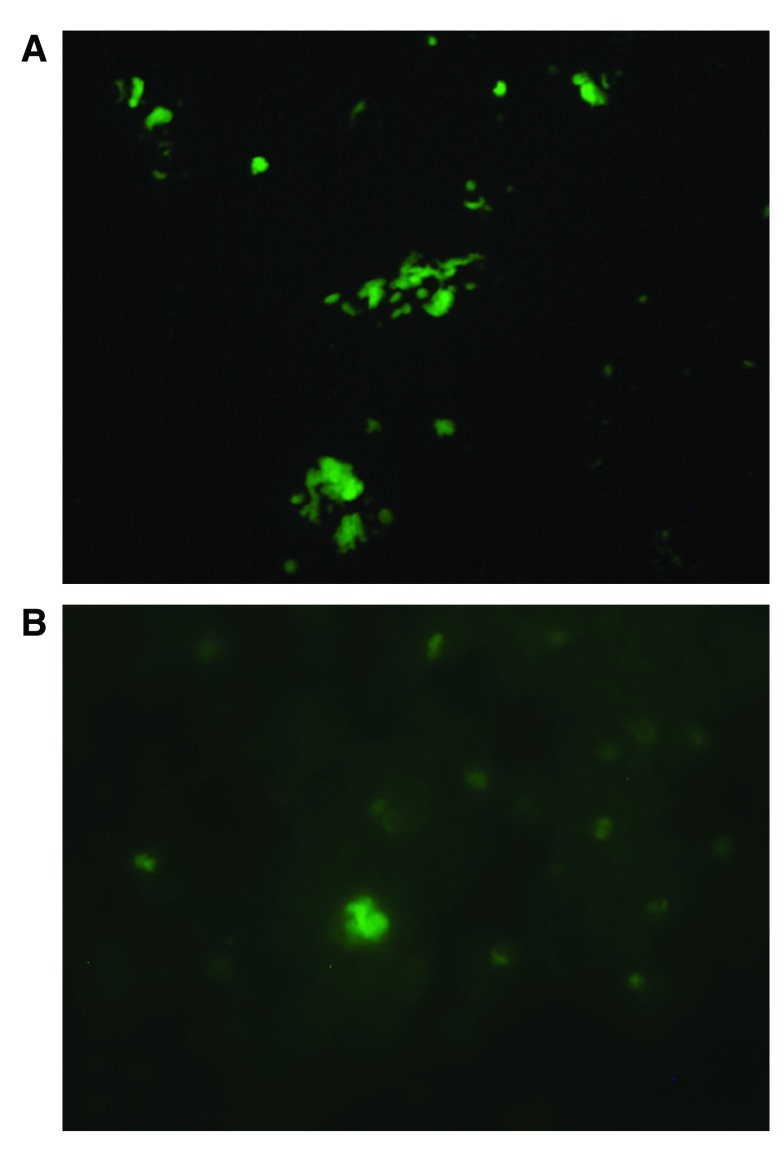
**A**: Molecular hybridization of *B. burgdorferi*-specific FlaB probe with seminal culture from Patient 6. Note intensely staining spiral and round forms in culture. 400× magnification. **B**: Molecular hybridization of *B. burgdorferi*-specific FlaB probe with vaginal culture from Patient 7. Note intensely staining spiral and round forms in culture. 400× magnification. See Dataset, data file 4.

Hybridization with the Fla B probe was positive for genital culture pellets from Patients 2–9 ( Table 4). The culture pellet from Patient 1 was lost during processing. The molecular probe showed intense staining in vaginal secretions and less intense staining in semen samples ( Figure 3A and 3B). See Dataset, data file 4.

### 5. PCR testing

***A. Australian Biologics.*** *Borrelia* 16S rRNA sequence was not detected by real-time PCR in any of the control genital culture pellets. In contrast, *Borrelia* 16S rRNA sequence was detected in genital culture pellets from 11 of 13 patients ( Table 5A). Patient 2 had equivocal test results and Patient 3 had negative test results in seminal cultures. See Dataset, data file 5. Real-time PCR failed to detect treponemal gene sequences in any of the control or patient genital culture pellets. See Dataset, data file 5a. The 16S rRNA isolates from six patients were sequenced and subjected to BLAST analysis (see below).

**Table 5.** A: Real time PCR testing of genital culture concentrates performed by Australian Biologics. ND, not done. See Dataset, data file 5. B: Real time and nested PCR testing of blood and genital culture concentrates performed by University of New Haven. See Dataset, data file 7. ND, not done. **Table 5A: Real-time PCR – Australian Biologics.**

Control number	Genital culture – Real- time *Borrelia* PCR	Genital culture – Real- time *T. pallidum* PCR	Genital culture – Real- time *T. denticola* PCR
1 (M) seminal	Negative	Negative	Negative
2 (M) seminal	Negative	Negative	Negative
3 (F) vaginal	Negative	Negative	Negative
4 (F) vaginal	Negative	Negative	Negative

**Patient #** **sample**	**Genital culture – Real-** **time *Borrelia* PCR**	**Genital culture – Real-** **time *T. pallidum* PCR**	**Genital culture – Real-** **time *T. denticola* PCR**
1 (F) vaginal	positive (sequenced 99% match B-31)	negative	negative
2 (M) seminal	equivocal	negative	negative
3 (M) seminal	negative	negative	negative
4 (F) vaginal	positive	negative	negative
5 (F) vaginal	positive	negative	negative
6 (M) seminal	positive (sequenced 100% match B-31)	negative	negative
7 (F) vaginal	positive (sequenced 98% match B-31)	negative	negative
8 (M) seminal	positive	negative	negative
9 (F) vaginal	positive	negative	negative
10 (M) seminal	positive (sequenced 100% match YOR)	ND	ND
11 (F) vaginal	positive (sequenced 100% match YOR)	ND	ND
12 (M) seminal	positive	ND	ND
13 (F) vaginal	positive (sequenced 100% match B-31)	ND	ND

***B. University of New Haven.*** PCR testing using the TaqMan assay for *Borrelia* 16S rRNA sequence was positive in blood culture pellets from seven of nine patients tested ( Table 5B). Patients 1 and 5 had negative results in blood culture pellets using the TaqMan assay, but both were positive by nested PCR for the pyrG gene. In addition, nested PCR targeting the fla gene was performed on blood culture pellets from Patients 2, 3 and 4, and nested PCR targeting the 16S rRNA gene was performed on the blood culture pellet from Patient 6. The samples were positive, and sequencing revealed 99–100% homology with Bb *sensu stricto* strain B-31 ( Table 5B). See Dataset, data file 7.

**Table:** **Table 5B: PCR – University of New Haven.**

Control number	Blood culture	Genital culture – 16S rRNA Taq Man PCR	Genital culture – Nested PCR
1 (M)	ND	Negative	pyrG negative, fla negative
2 (M)	ND	Negative	pyrG negative, fla negative
3 (F)	ND	Negative	pyrG negative, fla negative
4 (F)	ND	Negative	pyrG negative, fla negative

**Patient number**	**Blood culture – primers** **with positive detection**		**Genital culture – primers** **with positive detection**
1 (F)	pyrG		16S rRNA Taq Man
2 (M)	16S rRNA Taq Man, fla (sequenced, 100% match B-31)		16S rRNA Taq Man
3 (M)	16S rRNA Taq Man, fla (sequenced, 100% match B-31)		16S rRNA Taq Man, fla, 16S rRNA (sequenced, 99% match B-31)
4 (F)	16S rRNA Taq Man, fla (sequenced, 99% match B-31)		16S rRNA Taq Man
5 (F)	pyrG		16S rRNA Taq Man
6 (M)	16S rRNA Taq Man 16S rRNA (sequenced, 99% match B31)		16S rRNA
7 (F)	16S rRNA Taq Man, pyrG		16S rRNA Taq Man, 16S rRNA, fla
8 (M)	16S rRNA Taq Man		16S rRNA Taq Man
9 (F)	16S rRNA Taq Man		16S rRNA Taq Man
10 (M)	ND		ND
11 (F)	ND		ND
12 (M)	ND		pyrG (sequenced, 99% match B-31)
13 (F)	ND		ND

PCR testing using the TaqMan assay for *Borrelia* 16S rRNA sequence was negative in all four control genital culture pellets, and nested PCR targeting the pyrG and fla genes was negative in all four control samples, confirming the results of the TaqMan assay ( Table 5B). In contrast, eight of nine patients were positive for TaqMan 16S rRNA sequence in the genital culture pellets. Patient 6 was negative using the TaqMan assay for 16S rRNA sequence but positive using nested PCR targeting a different portion of the 16S rRNA gene ( Table 5B). Nested PCR targeting the fla gene (Patient 3) and the 16S rRNA gene (Patients 3 and 7) was also performed on genital culture pellets and was positive in those patients, confirming the results of the TaqMan assay. Patient 12 had positive PCR targeting the pyrG gene with confirmatory sequencing (see below).

### 6. Sequencing of *Borrelia* detected in blood and genital cultures

PCR isolates of the vaginal culture from Patient 1 (Australian Biologics) and the seminal culture from Patient 3 (University of New Haven) were subjected to Sanger sequencing and BLAST analysis and showed 97–99% homology with Bb *sensu stricto* strain B-31 ( Table 5A and Table 5B). See Datasets, data files 6 and 7. PCR isolates of blood cultures from Patients 2, 3, 4 and 6 were subjected to Sanger sequencing and BLAST analysis at University of New Haven and showed 99–100% homology with Bb *sensu stricto* strain B-31 ( Table 5B). See Dataset, data file 7.

PCR isolates of genital cultures from three couples having unprotected sex (Patients 6–7, 10–11 and 12–13) were subjected to Sanger sequencing and BLAST analysis. Patients 6, 7, 10, 11 and 13 had sequencing done at Australian Biologics, while Patient 12 had sequencing done at University of New Haven. Sequencing revealed that the first and third couples had *Borrelia* strains that matched Bb *sensu stricto* strain B-31 ( Table 6). In contrast, the second couple had PCR sequences that matched *B. hermsii* strain YOR. Thus the *Borrelia* strain shared by this couple differed significantly from the strains identified in the other couples. See Dataset, data file 6.

**Table 6.** Comparison of seminal and vaginal *Borrelia* gene sequences using BLAST analysis. Sequencing for Patients 6, 7, 10, 11 and 13 was done at Australian Biologics. Sequencing for Patient 12 was done at University of New Haven. See Dataset, data file 6.

Patient	Description	Maximum Score	Total Score	Query Cover	E Value	Reference Strain Match
6 (M)	Bb sensu stricto (B31)	230	230	84%	3e-57	100%
7 (F)	Bb sensu stricto (B31)	224	224	83%	2e-55	98%
10 (M)	B. hermsii (YOR)	32.2	1229	75%	1.5	100%
11 (F)	B. hermsii (YOR)	30.2	599	84%	2.1	100%
12 (M)	Bb sensu stricto (B31)	1218	1218	95%	1e-63	99%
13 (F)	Bb sensu stricto (B31)	97.6	4880	87%	1e-20	100%

Updated data of *Borrelia* spirochetes in human vaginal and seminal secretions The dataset contains data files 1, 2, 2a, 3, 3a, 4, 5, 5a, 6 and 7. Detailed legends for each files can be found in the text file provided. Click here for additional data file. Copyright: © 2015 Middelveen MJ et al. 2015 Data associated with the article are available under the terms of the Creative Commons Zero "No rights reserved" data waiver (CC0 1.0 Public domain dedication).

## Discussion

In this study using standard and published culture, immunohistochemical, molecular hybridization and PCR techniques, we have shown that *Borrelia* strains are present in semen and vaginal secretions from patients with Lyme disease. Simultaneous testing for treponemal spirochetes was negative in genital secretions of all Lyme disease patients, confirming the specificity of *Borrelia* detection in these patients. Furthermore we have shown that couples having unprotected sex have virtually identical strains of *Borrelia* in their genital secretions, suggesting that *Borrelia* spirochetes might be transmitted from person to person without a tick vector.

As expected, PCR sequencing of cultured *Borrelia* from semen and vaginal secretions yielded primarily Bb *sensu stricto* strains, reflecting the North American origin of our study subjects. In addition, PCR sequencing of genital secretions from one couple yielded identical strains of Bb *sensu stricto* strains in two different laboratories. However, we were surprised to find one couple with identical strains of *B. hermsii* in their genital secretions. The presence of a distinct *Borrelia* strain in semen and vaginal secretions from a sexually active couple that differs from strains found in other couples supports the premise of *Borrelia* transmission via shared genital secretions. The finding is analogous to sharing distinct human immunodeficiency virus (HIV) strains, which is well recognized in sexual partners with HIV/AIDS ( Shaw & Hunter, 2012).

Animal models have provided compelling evidence for contact transmission of Bb without a tick vector in mice, ducks, cats and dogs ( Burgess *et al.*, 1986; Burgess & Patrican, 1987; Burgess, 1989; Burgess, 1992; Wright & Neilsen, 1990). Bb has been shown to survive in stored semen from dogs, rams and bulls ( Kumi-Diaka & Harris, 1995). Furthermore, seminal transmission of Bb has been noted in dogs, as described above ( Gustafson, 1993). In contrast, contact transmission of Bb could not be demonstrated in Lewis rats and Syrian golden hamsters ( Moody & Barthold, 1991; Woodrum & Oliver, 1999). Technical limitations in the study of these highly inbred rodents including limited contact between animals and failure to perform molecular testing may have contributed to the negative results.

While it is not possible to perform controlled sexual transmission studies of *Borrelia* in humans, several investigators have speculated that this mode of transmission is possible ( Bach, 2001; Harvey & Salvato, 2003; Stricker *et al.*, 2004). The suggestion that Bb could be transmitted sexually was initially proposed by Bach in 2001. He observed that sexually active patients had a marked propensity for antibiotic failure and speculated that re-infection occurred by intimate person-to-person contact. Bb DNA was detected by PCR technology in human breast milk, umbilical cord blood, semen and vaginal secretions taken from patients presenting at his practice ( Bach, 2001).

The study of a group of chronically ill Bb-seropositive and PCR-positive patients in Houston, Texas – a non-endemic area – provided epidemiological evidence that Lyme disease could spread in the absence of a suitable vector ( Harvey & Salvato, 2003). In the absence of infected ticks, intimate person-to-person transfer was implicated as the probable means of transmission ( Harvey & Salvato, 2003). A study by Stricker *et al.* provided clinical and immunological evidence for Bb transmission from partner to partner. In heterosexual seropositive couples with Lyme disease in which only one partner had a documented tick bite, the partner with the documented tick bite tended to have more severe clinical manifestations of the disease and a lower CD57 natural killer (NK) cell level ( Stricker *et al.*, 2004). This difference in clinical severity and CD57 NK cell level was not noted in seropositive couples diagnosed with Lyme disease in which both partners had a documented history of tick bite ( Stricker *et al.*, 2004). Sexual transfer of *Borrelia* infection through mucosal contact therefore seems possible in humans. The fact that we have been able to culture motile, actively reproducing, viable spirochetes from human genital secretions supports this hypothesis.

Recent reports from the Centers for Disease Control and Prevention (CDC) indicate that more than 300,000 cases of Lyme disease are diagnosed yearly in the USA ( CDC, 2013). Sexual transmission of *Borrelia* may partly explain the large number of annual cases that is almost two times higher than breast cancer and six times higher that HIV/AIDS ( Stricker & Johnson, 2014). Recognition of possible sexual transmission of *Borrelia* in both humans and animals is fundamentally important because of the epidemiological implications. If sexual transmission of *Borrelia* occurs in both animals and humans, this mode of transmission is a possible means of introducing *Borrelia* infection into areas not considered endemic and of introducing the spirochete to new reservoirs. *Borrelia* would also join the list of other spirochetes that are either proven or postulated to be sexually transmitted, including the spirochetal agents of syphilis and leptospirosis ( Harrison & Fitzgerald, 1988; Maatouk & Moutran, 2014). Of note, sexual transmission of other tickborne agents in animals and humans has also been proven or postulated ( Facco *et al.*, 1992; Kruszewska & Tylewska-Wierzbanowska, 1993; Metcalf, 2001; Miceli *et al.*, 2010; Milazzo *et al.*, 2001).

The number of spirochetes needed to infect an animal or human varies according to strain-specific biological and transmission factors. In mouse studies of experimental *Borrelia* infection, the 50% infectious dose was 18 spirochetes with tick salivary gland extract and 251 spirochetes with tick midgut extract ( Cook, 2014). Transmission studies of syphilis using “human volunteers” found that the 50% infectious dose was approximately 57 organisms ( LaFond & Lukehart, 2006). At present, the spirochetal load in genital secretions from Lyme disease patients is unknown, but it appears that genital infection could be induced by a relatively small number of organisms based on the studies outlined above. It is known that seminal plasma inhibits the immune response to Gram-negative pathogens ( Brooks *et al.*, 1981), while the female genital tract induces immune factors that may be conducive to spirochete survival ( Clark & Schust, 2013; Wira *et al.*, 2005). The role of the male and female genital tracts in tolerance and propagation of *Borrelia* infection merits further study.

Lyme disease diagnosis is based largely upon serological testing using CDC-sanctioned two-tier surveillance criteria supported by FDA-approved commercial test kits. While most patients in this study did have positive serological test results for Lyme borreliosis, some were considered serologically negative, and the majority of our study subjects did not meet the positive standard as defined by the CDC surveillance criteria ( CDC, 2014a). We were able to detect *Borrelia* spirochetes in the blood and/or genital secretions of all patients who were clinically diagnosed with Lyme disease, demonstrating that the CDC surveillance protocol is inadequate diagnostically. Inadequate diagnostic methodology undoubtedly results in under-reporting of Lyme disease, and at least one group has speculated that this substandard methodology is considered acceptable because *Borrelia* is not sexually transmitted ( Lange & Sayyedi, 2002). In addition, if *Borrelia* spirochetes were transmitted sexually, then patients with false-negative results may unknowingly spread the infection to sexual partners.

The 2011 CDC case definition for Lyme disease states that a positive Bb culture confirms the diagnosis of the disease ( CDC, 2014b). Although culture of *Borrelia* genital isolates may be a useful diagnostic laboratory methodology in the future, detecting and characterizing cultured *Borrelia* isolates is not straightforward, and both false-positive and false-negative results could occur. In our experience, human clinical isolates from genital secretions frequently propagate prolifically in culture, but on occasion they do not. In such instances, the culture must be concentrated and specific staining should be conducted to ascertain the presence of spirochetes. Once detected, spirochetes must be characterized genetically for specific identification. PCR is currently the most reliable means for correctly identifying cultured isolates, but even this methodology has drawbacks and limitations ( Lange & Sayyedi, 2002; Nolte, 2012).

There are currently no standardized FDA-approved PCR protocols or kits available for Bb detection, so commercial PCR testing constitutes an array of “home brew” assays using different methodologies such as real-time PCR and nested PCR, with various primers targeting different genes, yielding wide differences in sensitivity and specificity ( Nolte, 2012; Schmidt, 1997; Yang *et al.*, 2012). False negatives can result because primers may be strain-specific and may not detect all *Borrelia* genotypes, and fluids such as blood, semen and vaginal secretions may contain substances inhibitory to the PCR process ( Lange & Sayyedi, 2002; Nolte, 2012; Yang *et al.*, 2012). The potential for false-positive PCR testing may also arise if there is DNA contamination in the laboratory, and appropriate positive and negative controls must be included in the assay ( Lange & Sayyedi, 2002; Nolte, 2012). We experienced differences in primer specificity in our clinical isolates and also found that inhibition occurred, particularly in semen cultures.

Another complicating factor in *Borrelia* isolation is the morphological variation of the spirochete, which includes spherical, granular or cystic forms. Morphological variants of Bb, some of which are not culturable, are well documented in the medical literature ( Barthold *et al.*, 2010; Hodzic *et al.*, 2014; Kurtti *et al.*, 1987; MacDonald, 2013; Meriläinen *et al.*, 2015; Mursic *et al.*, 1996). These variants may play a role in infection, enabling Bb and other pathogenic spirochetes to evade the immune system ( Döpfer *et al.*, 2012; Menten-Dedoyart *et al.*, 2012; Mursic *et al.*, 1996). Limited Bb growth and non-spiral morphology are thought to be induced by unfavorable environmental conditions ( Brorson *et al.*, 2009), and these features appear to be consistent with our observations. We found that *Borrelia* growth was more vigorous with more long slender morphological variants in cultures of genital secretions compared to cultures of blood, and we speculate that the human circulatory system is a more hostile environment for *Borrelia* than the human reproductive system.

The possibility of *Borrelia* contamination yielding false-positive PCR results in blood cultures from Lyme disease patients has been suggested ( Sapi *et al.*, 2013). This possibility is highly unlikely in our cultures of genital secretions for the following reasons: first, no reference strains of *Borrelia* that could cause contamination were present in the laboratory where cultures were performed. Second, the sequenced *Borrelia* strains were not 100% identical to the reference strains of *Borrelia*, implying that they were distinct from potentially contaminating reference strains. Third, testing was performed in three independent laboratories, and it would be highly unlikely to have contamination in all three locations. Fourth, negative controls were run with the molecular samples in the three independent laboratories, and the controls were consistently negative. Fifth, as noted above, one couple had a distinct strain of *Borrelia* in their genital secretions, so that selective contamination with two different reference strains would have had to occur in the PCR samples. Thus laboratory contamination yielding false-positive PCR results for *Borrelia* strains in the genital secretions is highly unlikely.

Several questions have been raised about the likelihood of *Borrelia* sexual transmission ( Craig, 2014). First, according to the CDC surveillance system Lyme disease occurs most commonly in children and older adults. However, the CDC surveillance system only captures about 10% of Lyme disease patients, and the other 90% may have a different demographic distribution consistent with sexual transmission, as shown in a recent study from Australia ( Mayne, 2015). A study from military treatment facilities in the USA “unexpectedly” found no association between the incidence of Lyme disease and the prevalence of infected ticks, and the rate of Lyme disease was 2.6 times higher in officers than enlisted men ( Rossi *et al.*, 2015). Second, while sexually transmitted diseases like herpes simplex virus (HSV) and gonorrhea show an urban predominance, Lyme disease has a more rural distribution ( Craig, 2014). However, Lyme disease is acquired in more ways than HSV and gonorrhea, and the rate of sexual transmission is unknown at present. Thus the epidemiology of Lyme disease may differ from other sexually transmitted diseases based on these undefined variables. Third, the transmission of HIV can be traced from one sex partner to another using HIV strain typing. Based on our study, a similar transmission pattern using *Borrelia* strain typing may be seen once larger studies are performed among couples having unprotected sex. In summary, sexual transmission of *Borrelia* is plausible in light of our limited knowledge about the risk of acquiring Lyme disease.

In conclusion, we have shown that *Borrelia* spirochetes are present in semen and vaginal secretions of patients with Lyme disease. Furthermore, virtually identical strains of *Borrelia* are present in couples having unprotected sex, suggesting that transmission via intimate contact without a tick vector may occur. The epidemiology and clinical risk of *Borrelia* sexual transmission remain to be determined.

## Data availability

The data referenced by this article are under copyright with the following copyright statement: Copyright: © 2015 Middelveen MJ et al.

Data associated with the article are available under the terms of the Creative Commons Zero "No rights reserved" data waiver (CC0 1.0 Public domain dedication). 

F1000Research: Dataset 1. Updated data of *Borrelia* spirochetes in human vaginal and seminal secretions., 10.5256/f1000research.5778.d46058 ( Middelveen *et al.*, 2015).

## Consent

Written informed consent to publish clinical details and study results was obtained from each participant.
