# Supplementary material for: Culture and identification of Borrelia spirochetes in human vaginal and seminal secretions
Source: F1000Res. 2015 Apr 27;3:309. Originally published 2014 Dec 18. [Version 3] doi: 10.12688/f1000research.5778.3 (PMC5482345; doi:10.12688/f1000research.5778.3)
Supplement: Updated data of Borrelia spirochetes in human vaginal and seminal secretions — The dataset contains data files 1, 2, 2a, 3, 3a, 4, 5, 5a, 6 and 7. Detailed legends for each files can be found in the text file provided. [file f1000research-3-6856-s0000.tgz › 3a95801b-ea1b-4731-9d1e-5376b3b8a9eb_Data_new.zip!/Data 5a/Control2 Trep.pdf]

AUSTRALIAN BIOLOGICS
TESTING SERVICES P/L
ABN 83 057 347 387

LABORATORY REPORT

Suite 605, 6th Flr
379-383 Pitt St
SYDNEY NSW 2000
Ph: (02) 9283 0807
Fx: (02) 9283 0910

REFERRING PRACTITIONER
Raphael Stricker

Phone: Fax:

PATIENT
Name: Surname:
Address:

DOB: F ☐ Collection Date: 25/11/13
Test #: 1 M ☐ AmendedReport Date: 28/11/14

MOLECULAR DIAGNOSTICS OF INFECTIOUS DISEASES

LAB Number: CK1193

Specimen: Semen

Tests performed	Result
Treponema denticola	Not Detected
Treponema pallidum	Not Detected

RESULTS

Result Interpretation:

- Detected: Indicates that the DNA of the organism has been detected in this sample.
- Not Detected: Indicates that no DNA of the organism has been detected in this sample.
- Equivocal: Indicates the assay has given 2 positive and 2 negative result for this sample.
- Inhibited: Indicates the presence of PCR inhibitors in this sample which reduces the sensitivity of the assay leading to false-negative results.

J Burke
(Director)

Disclaimer: The following disclaimer should be made:
a) Diagnosis should not be based on laboratory test alone. Results should be interpreted in conjunction with clinical symptoms and patient history.

Lower Limit of Detection for PCR levels

	Genbank Accession No.	Method	Lower Limits of Detection
Mycoplasma species	AP012303.1/ CP003773.1	Nested PCR	0.01µg/µL
Mycoplasma fermentans	CP002458.1	Nested PCR	0.1 µg/µL
Chlamydia trachomatis	X06707.3	Real-Time PCR	16 copies/ µL
Chlamydophila pneumoniae	GQ918216.1	Real-Time PCR	16 copies/ µL
Borrelia species	CP002228.1	Culture and/or Real-Time PCR	0.01 µg/µL
