# Supplementary material for: Culture and identification of Borrelia spirochetes in human vaginal and seminal secretions
Source: F1000Res. 2015 Apr 27;3:309. Originally published 2014 Dec 18. [Version 3] doi: 10.12688/f1000research.5778.3 (PMC5482345; doi:10.12688/f1000research.5778.3)
Supplement: Updated data of Borrelia spirochetes in human vaginal and seminal secretions — The dataset contains data files 1, 2, 2a, 3, 3a, 4, 5, 5a, 6 and 7. Detailed legends for each files can be found in the text file provided. [file f1000research-3-6856-s0000.tgz › 3a95801b-ea1b-4731-9d1e-5376b3b8a9eb_Data_new.zip!/Data 5a/Patient1 Trep.pdf]

AUSTRALIAN BIOLOGICS
TESTING SERVICES P/L
ABN 83 057 347 387

LABORATORY REPORT

Suite 605, 6th Flr
379-383 Pitt St
SYDNEY NSW 2000
Ph: (02) 9283 0807
Fx: (02) 9283 0910

REFERRING PRACTITIONER		PATIENT	
Marianne Middelveen		Name:	Surname:
		Address:	
		DOB:	F ☐ Collection Date:
Ph:	Fax:	Test #: 1	M ☐ Report Date: 3/1/14

MOLECULAR BIOLOGY

LAB Number: CJ1558

Specimen: Vaginal

Tests performed	Result	Genbank Accession No.	Method
Treponema denticola	Not Detected	JF700283.1	Real-Time
Treponema pallidum	Not Detected	CP004010.2	Real-Time

RESULTS

Disclaimer: The following disclaimer should be made:
a) Diagnosis should not be based on laboratory test alone. Results should be interpreted in conjunction with clinical symptoms and patient history.
