# Supplementary material for: Culture and identification of Borrelia spirochetes in human vaginal and seminal secretions
Source: F1000Res. 2015 Apr 27;3:309. Originally published 2014 Dec 18. [Version 3] doi: 10.12688/f1000research.5778.3 (PMC5482345; doi:10.12688/f1000research.5778.3)
Supplement: Updated data of Borrelia spirochetes in human vaginal and seminal secretions — The dataset contains data files 1, 2, 2a, 3, 3a, 4, 5, 5a, 6 and 7. Detailed legends for each files can be found in the text file provided. [file f1000research-3-6856-s0000.tgz › 3a95801b-ea1b-4731-9d1e-5376b3b8a9eb_Data_new.zip!/Data 5/Control4 PCRA.pdf]

AUSTRALIAN BIOLOGICS
TESTING SERVICES P/L
ABN 83 057 347 387

# LABORATORY REPORT

Suite 605, 6th Flr
379-383 Pitt St
SYDNEY NSW 2000
Ph: (02) 9283 0807
Fx: (02) 9283 0910

| REFERRING PRACTITIONER | | PATIENT | |
|---|---|---|---|
| Hilary Schlinger | | Name: | Surname: |
| | | Address: | |
| | | DOB: 29/10/70 | F ✓ Collection Date: 4/12/13 |
| Phone: | Fax: | Test #: 1 | M ☐ Report Date: 20/1/14 |

## MOLECULAR DIAGNOSTICS OF INFECTIOUS DISEASES

LAB Number: CK1487

Specimen: Vag Swab(fluid in vial)

| Tests performed | Result |
|---|---|
| Borrelia species | Not Detected |

RESULTS

Result Interpretation:

- Detected: Indicates that the DNA of the organism has been detected in this sample.
- Not Detected: Indicates that no DNA of the organism has been detected in this sample.
- Equivocal: Indicates the assay has given 2 positive and 2 negative result for this sample.
- Inhibited: Indicates the presence of PCR inhibitors in this sample which reduces the sensitivity of the assay leading to false-negative results.

Please Note: A negative result by PCR does not exclude the possibility of a low level presence of infection.

(Director)

Disclaimer: The following disclaimer should be made:
a) Diagnosis should not be based on laboratory test alone. Results should be interpreted in conjunction with clinical symptoms and patient history.

| | Genbank Accession No. | Method | Lower Limits of Detection |
|---|---|---|---|
| Mycoplasma species | AP012303.1/ CP003773.1 | Nested PCR | 0.01µg/µL |
| Mycoplasma fermentans | CP002458.1 | Nested PCR | 0.1 µg/µL |
| Chlamydia trachomatis | X06707.3 | Real-Time PCR | 16 copies/ µL |
| Chlamydophila pneumoniae | GQ918216.1 | Real-Time PCR | 16 copies/ µL |
| Borrelia species | CP002228.1 | Culture and/or Real-Time PCR | 0.01 µg/µL |
