# Supplementary material for: Culture and identification of Borrelia spirochetes in human vaginal and seminal secretions
Source: F1000Res. 2015 Apr 27;3:309. Originally published 2014 Dec 18. [Version 3] doi: 10.12688/f1000research.5778.3 (PMC5482345; doi:10.12688/f1000research.5778.3)
Supplement: Updated data of Borrelia spirochetes in human vaginal and seminal secretions — The dataset contains data files 1, 2, 2a, 3, 3a, 4, 5, 5a, 6 and 7. Detailed legends for each files can be found in the text file provided. [file f1000research-3-6856-s0000.tgz › 3a95801b-ea1b-4731-9d1e-5376b3b8a9eb_Data_new.zip!/Data 5/Patient2 PCRA.pdf]

AUSTRALIAN BIOLOGICS
TESTING SERVICES P/L
ABN 83 057 347 387

# LABORATORY REPORT

Suite 605, 6th Flr
379-383 Pitt St
SYDNEY NSW 2000
Ph: (02) 9283 0807
Fx: (02) 9283 0910

| REFERRING PRACTITIONER | | PATIENT | |
|---|---|---|---|
| Marianne Middelveen | | Name: | Surname: |
| | | Address: | |
| | | DOB: | F ☐ Collection Date: |
| Phone: | Fax: | Test #: 1 | M ☐ Report Date: 27/9/13 |

## MOLECULAR BIOLOGY

LAB Number: CJ1902

Specimen: Seminal

| Tests performed | Result | Genbank Accession No. | Method |
|---|---|---|---|
| Borrelia Seminal | Equivocal | Borr: (CP002228.1) | Culture/ Real-Time PCR |

RESULTS

Disclaimer: The following disclaimer should be made:
a) Diagnosis should not be based on laboratory test alone. Results should be interpreted in conjunction with clinical symptoms and patient history.
