# Supplementary material for: Culture and identification of Borrelia spirochetes in human vaginal and seminal secretions
Source: F1000Res. 2015 Apr 27;3:309. Originally published 2014 Dec 18. [Version 3] doi: 10.12688/f1000research.5778.3 (PMC5482345; doi:10.12688/f1000research.5778.3)
Supplement: Updated data of Borrelia spirochetes in human vaginal and seminal secretions — The dataset contains data files 1, 2, 2a, 3, 3a, 4, 5, 5a, 6 and 7. Detailed legends for each files can be found in the text file provided. [file f1000research-3-6856-s0000.tgz › 3a95801b-ea1b-4731-9d1e-5376b3b8a9eb_Data_new.zip!/Data 5/Patient3 PCRA.pdf]

AUSTRALIAN BIOLOGICS
TESTING SERVICES P/L
ABN 83 057 347 387

# LABORATORY REPORT

Suite 605, 6th Flr
379-383 Pitt St
SYDNEY NSW 2000
Ph: (02) 9283 0807
Fx: (02) 9283 0910

**REFERRING PRACTITIONER**
Marianne Middelveen

Phone:
Fax:

**PATIENT**
Name:
Surname:
Address:
DOB:
F ☐
Collection Date:
Test #: 1
M ☐
Report Date: 27/9/13

## MOLECULAR BIOLOGY

LAB Number: CJ1901

Specimen: Seminal

| Tests performed | Result | Genbank Accession No. | Method |
|---|---|---|---|
| Borrelia Seminal | Not Detected | Borr: (CP002228.1) | Culture/ Real-Time PCR |

RESULTS

Please Note: A negative result by PCR does not exclude the possibility of a low level presence of Borrelia.

Disclaimer: The following disclaimer should be made:
a) Diagnosis should not be based on laboratory test alone. Results should be interpreted in conjunction with clinical symptoms and patient history.
