# Supplementary material for: Culture and identification of Borrelia spirochetes in human vaginal and seminal secretions
Source: F1000Res. 2015 Apr 27;3:309. Originally published 2014 Dec 18. [Version 3] doi: 10.12688/f1000research.5778.3 (PMC5482345; doi:10.12688/f1000research.5778.3)
Supplement: Updated data of Borrelia spirochetes in human vaginal and seminal secretions — The dataset contains data files 1, 2, 2a, 3, 3a, 4, 5, 5a, 6 and 7. Detailed legends for each files can be found in the text file provided. [file f1000research-3-6856-s0000.tgz › 3a95801b-ea1b-4731-9d1e-5376b3b8a9eb_Data_new.zip!/Data 6/Patient10 AB.pdf]

AUSTRALIAN BIOLOGICS
TESTING SERVICES P/L
ABN 83 057 347 387

LABORATORY REPORT

Suite 605, 6th Flr
379-383 Pitt St
SYDNEY NSW 2000
Ph: (02) 9283 0807
Fx: (02) 9283 0910

REFERRING PRACTITIONER	PATIENT
Marianne Middelveen	Name: Surname:
	Address:
	DOB: F ☐ Collection Date:
Phone: Fax:	Test #: 1 M ☐ Report Date: 4/4/14

MOLECULAR DIAGNOSTICS OF INFECTIOUS DISEASES

LAB Number: CL0551

Specimen: Semen

Tests performed	Result
Borrelia	Detected

RESULTS

Result Interpretation:

- Detected: Indicates that the DNA of the organism has been detected in this sample.
- Not Detected: Indicates that no DNA of the organism has been detected in this sample.
- Equivocal: Indicates the assay has given 2 positive and 2 negative result for this sample.
- Inhibited: Indicates the presence of PCR inhibitors in this sample which reduces the sensitivity of the assay leading to false-negative results.

Please Note: A negative result by PCR does not exclude the possibility of a low level presence of infection.

J Burke
(Director)

Disclaimer: The following disclaimer should be made:
a) Diagnosis should not be based on laboratory test alone. Results should be interpreted in conjunction with clinical symptoms and patient history.

	Genbank Accession No.	Method	Lower Limits of Detection
Mycoplasma species	AP012303.1/ CP003773.1	Nested PCR	0.01µg/µL
Mycoplasma fermentans	CP002458.1	Nested PCR	0.1 µg/µL
Chlamydia trachomatis	X06707.3	Real-Time PCR	16 copies/ µL
Chlamydophila pneumoniae	GQ918216.1	Real-Time PCR	16 copies/ µL
Borrelia species	CP002228.1	Culture and/or Real-Time PCR	0.01 µg/µL

BLAST ®

Basic Local Alignment Search Tool

NCBI/ BLAST/ blastn suite/ **Formatting Results - YGGA5EVR014**

Formatting options

Download

Blast report description

CL551

**RID**	YGGA5EVR014 (Expires on 08-12 12:06 pm)
**Query ID**	lcl|40927
**Description**	None
**Molecule type**	nucleic acid
**Query Length**	160
**Database Name**	nr
**Description**	Nucleotide collection (nt)
**Program**	BLASTN 2.2.29+

Graphic Summary

Distribution of 200 Blast Hits on the Query Sequence

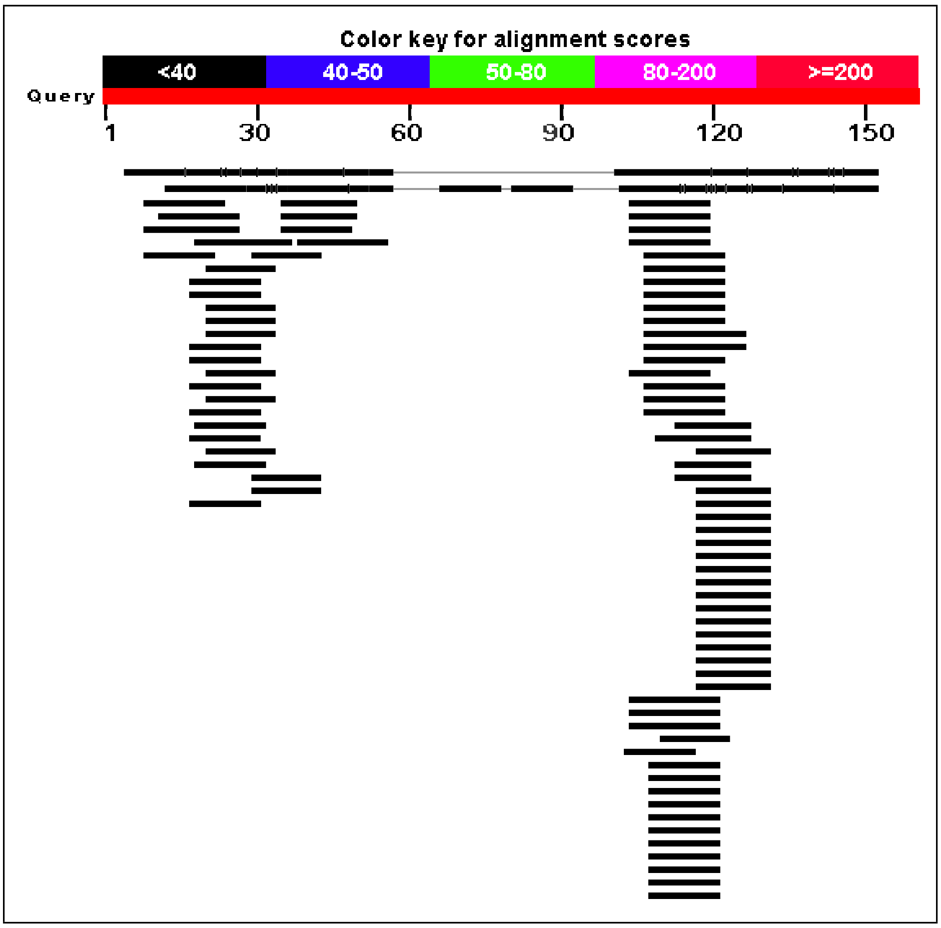

Descriptions

Sequences producing significant alignments:

Description	Max score	Total score	Query cover	E value	Ident	Accession
Borrelia garinii SZ, complete genome	32.2	1340	65%	1.5	100%	CP007564.1
Borrelia hermsii YOR, complete genome	32.2	1229	75%	1.5	100%	CP004146.1
Borrelia anserina BA2, complete genome	32.2	905	65%	1.5	100%	CP005829.1
Borrelia coriaceae Co53 plasmid Contig0029 genomic sequence	32.2	32.2	10%	1.5	100%	CP005773.1
Borrelia coriaceae Co53, complete genome	32.2	1055	65%	1.5	100%	CP005745.1
Borrelia hermsii YBT, complete genome	32.2	1162	70%	1.5	100%	CP005706.1
Borrelia hermsii MTW, complete genome	32.2	1229	75%	1.5	100%	CP005680.1
Borrelia burgdorferi CA382, complete genome	32.2	1451	75%	1.5	95%	CP005925.1
Borrelia afzelii HLJ01, complete genome	32.2	1484	68%	1.5	95%	CP003882.1
Borrelia garinii NMJW1, complete genome	32.2	1340	65%	1.5	95%	CP003866.1
Borrelia crocidurae str. Achema, complete genome	32.2	1354	66%	1.5	100%	CP003426.1
Borrelia garinii BgVir chromosome linear, complete sequence	32.2	1388	61%	1.5	95%	CP003151.1
Borrelia afzelii PKo, complete genome	32.2	1437	68%	1.5	95%	CP002933.1
Borrelia bissettii DN127, complete genome	32.2	1283	70%	1.5	95%	CP002746.1
Borrelia burgdorferi N40, complete genome	32.2	1419	75%	1.5	95%	CP002228.1
Borrelia burgdorferi JD1, complete genome	32.2	1496	75%	1.5	95%	CP002312.1
Borrelia burgdorferi ZS7, complete genome	32.2	1455	75%	1.5	95%	CP001205.1
Borrelia hermsii DAH, complete genome	32.2	1113	70%	1.5	100%	CP000048.1
Borrelia afzelii PKo, complete genome	32.2	1437	68%	1.5	95%	CP000395.1
Borrelia garinii PBi, complete genome	32.2	1291	65%	1.5	95%	CP000013.1
Borrelia burgdorferi B31, complete genome	32.2	1451	75%	1.5	95%	AE000783.1
Borrelia hermsii YOR plasmid Contig0023 genomic sequence	30.2	54.5	19%	5.8	100%	CP004168.1
Borrelia miyamotoi FR64b, complete genome	30.2	1201	67%	5.8	100%	CP004217.1
Borrelia anserina BA2 plasmid Contig0002 genomic sequence	30.2	54.5	19%	5.8	95%	CP005830.1
Borrelia hermsii MTW plasmid Contig0011 genomic sequence	30.2	78.8	29%	5.8	100%	CP005688.1
Borrelia garinii PBi plasmid lp54, complete sequence	30.2	30.2	9%	5.8	100%	CP000015.1
Borrelia coriaceae Co53 plasmid Contig0010 genomic sequence	30.2	58.5	11%	5.8	95%	CP005754.1
Borrelia hermsii strain HS1 plasmid lp174, complete sequence	30.2	202	25%	5.8	95%	HM008709.2
Borrelia duttonii Ly plasmid pl70, complete sequence	30.2	161	30%	5.8	100%	CP000990.1
Borrelia duttonii Ly, complete genome	30.2	1354	65%	5.8	100%	CP000976.1
Borrelia garinii dbpA gene for decorin binding protein A, strain PBol	30.2	30.2	9%	5.8	100%	AJ841686.1
Borrelia garinii dbpA gene for decorin binding protein A, strain TN	30.2	30.2	9%	5.8	100%	AJ841685.1
Borrelia bavariensis dbpA gene for decorin binding protein A, strain PMue	30.2	30.2	9%	5.8	100%	AJ841677.1
