# Supplementary material for: Culture and identification of Borrelia spirochetes in human vaginal and seminal secretions
Source: F1000Res. 2015 Apr 27;3:309. Originally published 2014 Dec 18. [Version 3] doi: 10.12688/f1000research.5778.3 (PMC5482345; doi:10.12688/f1000research.5778.3)
Supplement: Updated data of Borrelia spirochetes in human vaginal and seminal secretions — The dataset contains data files 1, 2, 2a, 3, 3a, 4, 5, 5a, 6 and 7. Detailed legends for each files can be found in the text file provided. [file f1000research-3-6856-s0000.tgz › 3a95801b-ea1b-4731-9d1e-5376b3b8a9eb_Data_new.zip!/Data 6/Patient11 AB.pdf]

AUSTRALIAN BIOLOGICS
TESTING SERVICES P/L
ABN 83 057 347 387

LABORATORY REPORT

Suite 605, 6th Flr
379-383 Pitt St
SYDNEY NSW 2000
Ph: (02) 9283 0807
Fx: (02) 9283 0910

REFERRING PRACTITIONER

Marianne Middelveen

Phone: Fax:

PATIENT

Name: Surname:
Address:

DOB: F ☐ Collection Date:
Test #: 1 M ☐ Report Date: 4/4/14

MOLECULAR DIAGNOSTICS OF INFECTIOUS DISEASES

LAB Number: CL0548

Specimen: Vaginal

Tests performed	Result
Borrelia	Detected

RESULTS

Result Interpretation:

- Detected: Indicates that the DNA of the organism has been detected in this sample.
- Not Detected: Indicates that no DNA of the organism has been detected in this sample.
- Equivocal: Indicates the assay has given 2 positive and 2 negative result for this sample.
- Inhibited: Indicates the presence of PCR inhibitors in this sample which reduces the sensitivity of the assay leading to false-negative results.

Please Note: A negative result by PCR does not exclude the possibility of a low level presence of infection.

J Burke
(Director)

Disclaimer: The following disclaimer should be made:
a) Diagnosis should not be based on laboratory test alone. Results should be interpreted in conjunction with clinical symptoms and patient history.

	Genbank Accession No.	Method	Lower Limits of Detection
Mycoplasma species	AP012303.1/ CP003773.1	Nested PCR	0.01µg/µL
Mycoplasma fermentans	CP002458.1	Nested PCR	0.1 µg/µL
Chlamydia trachomatis	X06707.3	Real-Time PCR	16 copies/ µL
Chlamydophila pneumoniae	GQ918216.1	Real-Time PCR	16 copies/ µL
Borrelia species	CP002228.1	Culture and/or Real-Time PCR	0.01 µg/µL

BLAST ®

Basic Local Alignment Search Tool

NCBI/ BLAST/ blastn suite/ **Formatting Results - XGMF0FC2014**
Formatting options
Download
Blast report description

CL548

**RID**	XGMF0FC2014 (Expires on 07-31 10:01 am)
**Query ID**	lcl|16203
**Description**	None
**Molecule type**	nucleic acid
**Query Length**	69
**Database Name**	nr
**Description**	Nucleotide collection (nt)
**Program**	BLASTN 2.2.29+

Graphic Summary

Distribution of 200 Blast Hits on the Query Sequence

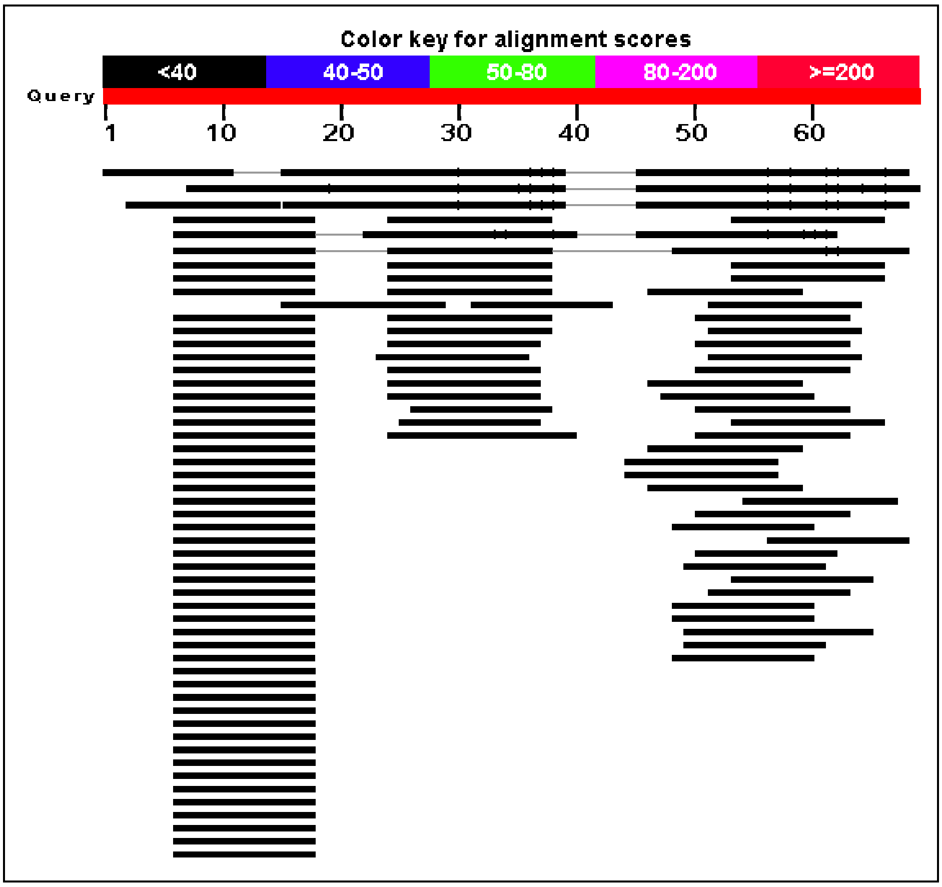

Descriptions

Sequences producing significant alignments:

Description	Max score	Total score	Query cover	E value	Ident	Accession
Borrelia hermsii YOR, complete genome	30.2	599	84%	2.1	100%	CP004146.1
Borrelia hermsii YBT, complete genome	30.2	605	81%	2.1	100%	CP005706.1
Borrelia hermsii MTW, complete genome	30.2	554	68%	2.1	100%	CP005680.1
Borrelia burgdorferi strain ACA-1 tRNA-guanine transglycosylase (tgt) gene, partial cds	28.2	28.2	20%	8.5	100%	KC416369.1
Borrelia afzelii HLJ01, complete genome	28.2	411	68%	8.5	100%	CP003882.1
Borrelia garinii BgVir chromosome linear, complete sequence	28.2	303	71%	8.5	100%	CP003151.1
Borrelia afzelii PKo, complete genome	28.2	388	68%	8.5	100%	CP002933.1
Borrelia afzelii PKo tRNA-guanine transglycosylase (tgt) gene, partial cds	28.2	28.2	20%	8.5	100%	EF537558.1
Borrelia garinii strain PBi tRNA-guanine transglycosylase (tgt) gene, partial cds	28.2	28.2	20%	8.5	100%	EF537557.1
Borrelia hermsii DAH, complete genome	28.2	668	79%	8.5	100%	CP000048.1
Borrelia afzelii PKo, complete genome	28.2	411	68%	8.5	100%	CP000395.1
Borrelia garinii PBi, complete genome	28.2	281	71%	8.5	100%	CP000013.1
Borrelia garinii SZ, complete genome	26.3	322	72%	33	100%	CP007564.1
Borrelia miyamotoi FR64b plasmid plasmid_31 genomic sequence	26.3	26.3	18%	33	100%	CP004248.1
Borrelia miyamotoi FR64b plasmid plasmid_12 genomic sequence	26.3	26.3	18%	33	100%	CP004229.1
Borrelia miyamotoi FR64b plasmid plasmid_02 genomic sequence	26.3	26.3	18%	33	100%	CP004219.1
Borrelia crocidurae DOU, complete genome	26.3	541	76%	33	100%	CP004267.1
Borrelia anserina BA2 plasmid Contig0010 genomic sequence	26.3	26.3	18%	33	100%	CP005838.1
Borrelia anserina BA2, complete genome	26.3	514	63%	33	100%	CP005829.1
Borrelia parkeri SLO, complete genome	26.3	676	68%	33	100%	CP005851.1
Borrelia coriaceae Co53 plasmid Contig0043 genomic sequence	26.3	26.3	18%	33	100%	CP005787.1
Borrelia coriaceae Co53 plasmid Contig0021 genomic sequence	26.3	26.3	18%	33	100%	CP005765.1
Borrelia parkeri HR1, complete genome	26.3	648	65%	33	100%	CP007022.1
Borrelia miyamotoi LB-2001, complete genome	26.3	524	68%	33	100%	CP006647.1
Borrelia burgdorferi CA382, complete genome	26.3	449	72%	33	100%	CP005925.1
Borrelia garinii NMJW1, complete genome	26.3	322	72%	33	100%	CP003866.1
Borrelia crocidurae str. Achema, complete genome	26.3	498	78%	33	100%	CP003426.1
Borrelia bissettii DN127, complete genome	26.3	435	73%	33	100%	CP002746.1
