# Supplementary material for: Culture and identification of Borrelia spirochetes in human vaginal and seminal secretions
Source: F1000Res. 2015 Apr 27;3:309. Originally published 2014 Dec 18. [Version 3] doi: 10.12688/f1000research.5778.3 (PMC5482345; doi:10.12688/f1000research.5778.3)
Supplement: Updated data of Borrelia spirochetes in human vaginal and seminal secretions — The dataset contains data files 1, 2, 2a, 3, 3a, 4, 5, 5a, 6 and 7. Detailed legends for each files can be found in the text file provided. [file f1000research-3-6856-s0000.tgz › 3a95801b-ea1b-4731-9d1e-5376b3b8a9eb_Data_new.zip!/Data 6/Patient12 UNH.pdf]

>P12_S_PYRG_PREMIX Sample_Name=P12_S_PYRG_PREMIX Chromat_id=3150347
>Read_id=3112242 Version=1 Length=692

NNNNNNNNNNNNNCTNNNAGNNTANTTAATAGCCATAATTTTACCTTCATATCCTTTGCCTCCAAAGCCGCCAGG
AACAATAATGCCGTCAAACTCTTTTAAACAGCTCTCATTTAAATCATTAGAATCAATTAAAGTGCTTTTAATAAG
CAAATCCAAATGGGCTGCAACATGAACCAAAGACTCTCTAATTGATGCATAAGAATCATCAAGTTCAGCATATTT
ACCACAAATAGCAATATTAATAATTTTTTTAGGCACAAAAAAATTAGATTTTATAACTCCTACAAGCTTTGAAAG
CTCTTCTATTTTTGGATCAACCTTAATATTTAACTTAGAGCTTAAAATCTCATGTACACCCTGCTTATAAAAAGA
TATAGGAATTTCATAAATAGTAGAAACATCAACATTGTCAATAATAGAAGTGCTCTCAACATTGCAAAACATTGC
CACTTTTTTTCTGATTTGGTCTGTCAATACTTGTGAACTTCTAGCAATAATTAAATCGGGGAAAATACCTGCTTT
ATTTAAGGTTTTAACACTTTGTTGAGTAGGTTTAGATTTTTGCTCATTAATTCCAGCTGGACTTGGCACATATGT
TAAATGAATAAAAGAAATATTACCACTCCCAATCTCCTGTCTTATTTGTCTTACTGTCTCAATAAATAAAATATT
TTCCATATCAANNNNNN

Score	1218 bits(659)
Expect	0.0
Identities	659/659(100%)
Gaps	0/659(0%)
Strand	Plus/Minus

Borrelia burgdorferi strain B31, complete genome
Sequence ID: gb|CP009656.1|Length: 910687Number of Matches: 1

Query 26
TTAATAGCCATAATTTTACCTTCATATCCTTTGCCTCCAAAGCCGCCAGGAACAATAATG 85
|||||||||||||||||||||||||||||||||||||||||||||||||||||||||||| Sbjct
589171 TTAATAGCCATAATTTTACCTTCATATCCTTTGCCTCCAAAGCCGCCAGGAACAATAATG
589112 Query 86
CCGTCAAACTCTTTTAAACAGCTCTCATTTAAATCATTAGAATCAATTAAAGTGCTTTTA 145
|||||||||||||||||||||||||||||||||||||||||||||||||||||||||||| Sbjct
589111 CCGTCAAACTCTTTTAAACAGCTCTCATTTAAATCATTAGAATCAATTAAAGTGCTTTTA
589052 Query 146
ATAAGCAAATCCAAATGGGCTGCAACATGAACCAAAGACTCTCTAATTGATGCATAAGAA 205
|||||||||||||||||||||||||||||||||||||||||||||||||||||||||||| Sbjct
589051 ATAAGCAAATCCAAATGGGCTGCAACATGAACCAAAGACTCTCTAATTGATGCATAAGAA
588992 Query 206
TCATCAAGTTCAGCATATTTACCACAAATAGCAATATTAATAA*ttttttt*AGGCAC*aaaa* 265
|||||||||||||||||||||||||||||||||||||||||||||||||||||||||||| Sbjct
588991 TCATCAAGTTCAGCATATTTACCACAAATAGCAATATTAATAATTTTTTTAGGCACAAAA
588932 Query 266
*aaa*TTAGATTTTATAACTCCTACAAGCTTTGAAAGCTCTTCTATTTTTGGATCAACCTTA 325
|||||||||||||||||||||||||||||||||||||||||||||||||||||||||||| Sbjct
588931 AAATTAGATTTTATAACTCCTACAAGCTTTGAAAGCTCTTCTATTTTTGGATCAACCTTA
588872 Query 326
ATATTTAACTTAGAGCTTAAAATCTCATGTACACCCTGCTTATAAAAAGATATAGGAATT 385
|||||||||||||||||||||||||||||||||||||||||||||||||||||||||||| Sbjct
588871 ATATTTAACTTAGAGCTTAAAATCTCATGTACACCCTGCTTATAAAAAGATATAGGAATT

588812 Query 386
TCATAAATAGTAGAAACATCAACATTGTCAATAATAGAAGTGCTCTCAACATTGCAAAAC 445
|||||||||||||||||||||||||||||||||||||||||||||||||||||||||||| Sbjct
588811 TCATAAATAGTAGAAACATCAACATTGTCAATAATAGAAGTGCTCTCAACATTGCAAAAC
588752 Query 446
ATTGCCACtttttttCTGATTTGGTCTGTCAATACTTGTGAACTTCTAGCAATAATTAAA 505
|||||||||||||||||||||||||||||||||||||||||||||||||||||||||||| Sbjct
588751 ATTGCCACTTTTTTTCTGATTTGGTCTGTCAATACTTGTGAACTTCTAGCAATAATTAAA
588692 Query 506
TCGGGGAAAATACCTGCTTTATTTAAGGTTTTAACACTTTGTTGAGTAGGTTTAGATTTT 565
|||||||||||||||||||||||||||||||||||||||||||||||||||||||||||| Sbjct
588691 TCGGGGAAAATACCTGCTTTATTTAAGGTTTTAACACTTTGTTGAGTAGGTTTAGATTTT
588632 Query 566
TGCTCATTAATTCCAGCTGGACTTGGCACATATGTTAAATGAATAAAAGAAATATTACCA 625
|||||||||||||||||||||||||||||||||||||||||||||||||||||||||||| Sbjct
588631 TGCTCATTAATTCCAGCTGGACTTGGCACATATGTTAAATGAATAAAAGAAATATTACCA
588572 Query 626
CTCCCAATCTCCTGTCTTATTTGTCTTACTGTCTCAATAAATAAAATATTTTCCATATC 684
||||||||||||||||||||||||||||||||||||||||||||||||||||||||||| Sbjct
588571 CTCCCAATCTCCTGTCTTATTTGTCTTACTGTCTCAATAAATAAAATATTTTCCATATC
588513
