# Supplementary material for: Culture and identification of Borrelia spirochetes in human vaginal and seminal secretions
Source: F1000Res. 2015 Apr 27;3:309. Originally published 2014 Dec 18. [Version 3] doi: 10.12688/f1000research.5778.3 (PMC5482345; doi:10.12688/f1000research.5778.3)
Supplement: Updated data of Borrelia spirochetes in human vaginal and seminal secretions — The dataset contains data files 1, 2, 2a, 3, 3a, 4, 5, 5a, 6 and 7. Detailed legends for each files can be found in the text file provided. [file f1000research-3-6856-s0000.tgz › 3a95801b-ea1b-4731-9d1e-5376b3b8a9eb_Data_new.zip!/Data 6/Patient13 AB.pdf]

AUSTRALIAN BIOLOGICS
TESTING SERVICES P/L
ABN 83 057 347 387

LABORATORY REPORT

Suite 605, 6th Flr
379-383 Pitt St
SYDNEY NSW 2000
Ph: (02) 9283 0807
Fx: (02) 9283 0910

REFERRING PRACTITIONER

Marianne Middelveen

Phone: Fax:

PATIENT

Name: Surname:
Address:

DOB: F ☐ Collection Date:
Test #: 1 M ☐ Report Date: 4/4/14

MOLECULAR DIAGNOSTICS OF INFECTIOUS DISEASES

LAB Number: CL0546

Specimen: Vaginal

Tests performed	Result
Borrelia	Detected

RESULTS

Result Interpretation:

- Detected: Indicates that the DNA of the organism has been detected in this sample.
- Not Detected: Indicates that no DNA of the organism has been detected in this sample.
- Equivocal: Indicates the assay has given 2 positive and 2 negative result for this sample.
- Inhibited: Indicates the presence of PCR inhibitors in this sample which reduces the sensitivity of the assay leading to false-negative results.

Please Note: A negative result by PCR does not exclude the possibility of a low level presence of infection.

J Burke
(Director)

Disclaimer: The following disclaimer should be made:
a) Diagnosis should not be based on laboratory test alone. Results should be interpreted in conjunction with clinical symptoms and patient history.

	Genbank Accession No.	Method	Lower Limits of Detection
Mycoplasma species	AP012303.1/ CP003773.1	Nested PCR	0.01µg/µL
Mycoplasma fermentans	CP002458.1	Nested PCR	0.1 µg/µL
Chlamydia trachomatis	X06707.3	Real-Time PCR	16 copies/ µL
Chlamydophila pneumoniae	GQ918216.1	Real-Time PCR	16 copies/ µL
Borrelia species	CP002228.1	Culture and/or Real-Time PCR	0.01 µg/µL

BLAST ®

Basic Local Alignment Search Tool

NCBI/ BLAST/ blastn suite/ **Formatting Results - YGG43AP8014**

Formatting options

Download

Blast report description

CL546

**RID**	YGG43AP8014 (Expires on 08-12 12:03 pm)
**Query ID**	lcl|21333
**Description**	None
**Molecule type**	nucleic acid
**Query Length**	70
**Database Name**	nr
**Description**	Nucleotide collection (nt)
**Program**	BLASTN 2.2.29+

Graphic Summary

Distribution of 200 Blast Hits on the Query Sequence

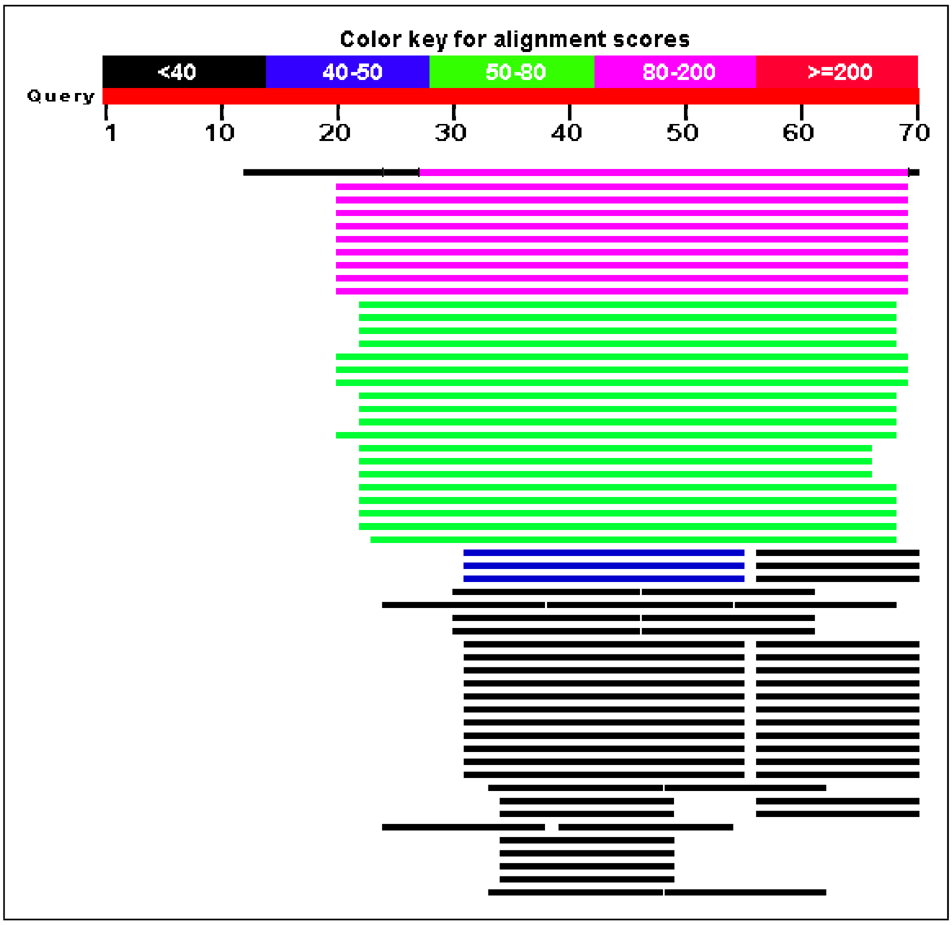

Descriptions

Sequences producing significant alignments:

Description	Max score	Total score	Query cover	E value	Ident	Accession
Borrelia burgdorferi CA382, complete genome	97.6	4880	87%	1e-20	100%	CP005925.1
Borrelia burgdorferi N40, complete genome	97.6	4787	87%	1e-20	100%	CP002228.1
Borrelia burgdorferi JD1, complete genome	97.6	4935	87%	1e-20	100%	CP002312.1
Borrelia burgdorferi ZS7, complete genome	97.6	4852	87%	1e-20	100%	CP001205.1
Borrelia burgdorferi B31, complete genome	97.6	4827	87%	1e-20	100%	AE000783.1
Borrelia garinii NMJW1, complete genome	89.7	5388	87%	3e-18	98%	CP003866.1
Borrelia garinii BgVir chromosome linear, complete sequence	89.7	5496	87%	3e-18	98%	CP003151.1
Borrelia bissettii DN127, complete genome	89.7	4899	87%	3e-18	98%	CP002746.1
Borrelia garinii PBi, complete genome	89.7	5649	90%	3e-18	98%	CP000013.1
Borrelia garinii SZ, complete genome	89.7	5388	87%	3e-18	98%	CP007564.1
Borrelia hermsii YOR, complete genome	75.8	4733	97%	4e-14	96%	CP004146.1
Borrelia anserina BA2, complete genome	75.8	4991	97%	4e-14	96%	CP005829.1
Borrelia hermsii MTW, complete genome	75.8	4773	97%	4e-14	96%	CP005680.1
Borrelia anserina Es nucleic acid enzyme (rpoC) gene, complete cds	75.8	75.8	65%	4e-14	96%	KJ136514.1
Borrelia afzelii HLJ01, complete genome	73.8	5342	87%	2e-13	94%	CP003882.1
Borrelia afzelii PKo, complete genome	73.8	5233	87%	2e-13	94%	CP002933.1
Borrelia afzelii PKo, complete genome	73.8	5209	87%	2e-13	94%	CP000395.1
Borrelia hermsii YBT, complete genome	67.9	4849	97%	1e-11	93%	CP005706.1
Borrelia miyamotoi LB-2001, complete genome	67.9	5311	97%	1e-11	93%	CP006647.1
Borrelia hermsii DAH, complete genome	67.9	4752	97%	1e-11	93%	CP000048.1
Borrelia coriaceae Co53, complete genome	63.9	5183	87%	2e-10	92%	CP005745.1
Borrelia parkeri HR1, complete genome	63.9	5041	97%	2e-10	93%	CP007022.1
Borrelia turicatae 91E135, complete genome	63.9	5006	87%	2e-10	93%	CP000049.1
Borrelia parkeri SLO, complete genome	63.9	4976	97%	2e-10	93%	CP005851.1
Borrelia crocidurae DOU, complete genome	60.0	5104	87%	2e-09	91%	CP004267.1
Borrelia recurrentis A1, complete genome	60.0	5341	87%	2e-09	91%	CP000993.1
Borrelia duttonii Ly, complete genome	60.0	5179	87%	2e-09	91%	CP000976.1
Borrelia miyamotoi FR64b, complete genome	60.0	5359	97%	2e-09	91%	CP004217.1
Borrelia crocidurae str. Achema, complete genome	58.0	5206	87%	1e-08	91%	CP003426.1
Borrelia afzelii strain VS461 30S ribosomal protein S10 gene, partial cds	40.1	40.1	34%	0.002	96%	EU363474.1
Borrelia afzelii strain A26S 30S ribosomal protein S10 gene, partial cds	40.1	40.1	34%	0.002	96%	EU363473.1
Borrelia afzelii ACA-1 strain ACA1 30S ribosomal protein S10 gene, partial cds	40.1	40.1	34%	0.002	96%	EU363470.1
Borrelia garinii BgVir plasmid lp54, complete sequence	32.2	453	55%	0.55	100%	CP003202.1
Borrelia valaisiana VS116 plasmid VS116_lp17, complete sequence	32.2	196	51%	0.55	100%	CP001439.1
Borrelia garinii Far04 plasmid Far04_lp54, complete sequence	32.2	427	55%	0.55	100%	CP001318.1
Borrelia garinii PBr plasmid PBr_lp54, complete sequence	32.2	404	55%	0.55	100%	CP001308.1
