# Supplementary material for: Culture and identification of Borrelia spirochetes in human vaginal and seminal secretions
Source: F1000Res. 2015 Apr 27;3:309. Originally published 2014 Dec 18. [Version 3] doi: 10.12688/f1000research.5778.3 (PMC5482345; doi:10.12688/f1000research.5778.3)
Supplement: Updated data of Borrelia spirochetes in human vaginal and seminal secretions — The dataset contains data files 1, 2, 2a, 3, 3a, 4, 5, 5a, 6 and 7. Detailed legends for each files can be found in the text file provided. [file f1000research-3-6856-s0000.tgz › 3a95801b-ea1b-4731-9d1e-5376b3b8a9eb_Data_new.zip!/Data 6/Patient6 AB.pdf]

AUSTRALIAN BIOLOGICS
TESTING SERVICES P/L
ABN 83 057 347 387

LABORATORY REPORT

Suite 605, 6th Flr
379-383 Pitt St
SYDNEY NSW 2000
Ph: (02) 9283 0807
Fx: (02) 9283 0910

REFERRING PRACTITIONER
Marianne Middelveen

Phone:
Fax:

PATIENT
Name:
Surname:
Address:

DOB:
F
Collection Date:
Test #:
M
Report Date: 13/9/13

MOLECULAR BIOLOGY

LAB Number: CJ1560

Specimen: Seminal

Tests performed	Result	Genbank Accession No.	Method
Borrelia Seminal	Detected	Borr: (CP002228.1)	Culture/ Real-Time PCR

RESULTS

Disclaimer: The following disclaimer should be made:
a) Diagnosis should not be based on laboratory test alone. Results should be interpreted in conjunction with clinical symptoms and patient history.

BLAST ®

Basic Local Alignment Search Tool

NCBI/ BLAST/ blastn suite/ **Formatting Results - 70NM613M01R**

Formatting options

Download

Blast report description

CJ1560

RID 70NM613M01R (Expires on 10-31 08:44 am)

**Query ID**	lcl|199869	**Database Name**	nr
**Description**	None	**Description**	Nucleotide collection (nt)
**Molecule type**	nucleic acid	**Program**	BLASTN 2.2.28+
**Query Length**	146		

Graphic Summary

Distribution of 9 Blast Hits on the Query Sequence

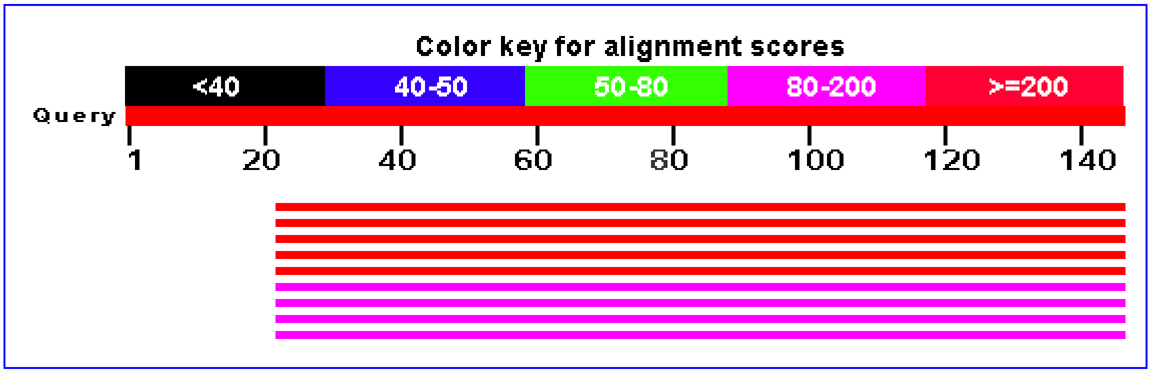

Descriptions

Description	Max score	Total score	Query cover	E value	Ident	Accession
Borrelia burgdorferi CA382, complete genome	230	230	84%	3e-57	100%	CP005925.1
Borrelia burgdorferi N40, complete genome	230	230	84%	3e-57	100%	CP002228.1
Borrelia burgdorferi ZS7, complete genome	230	230	84%	3e-57	100%	CP001205.1
Borrelia burgdorferi B31, complete genome	230	230	84%	3e-57	100%	AE000783.1
Borrelia burgdorferi JD1, complete genome	224	224	84%	1e-55	99%	CP002312.1
Borrelia garinii BgVir chromosome linear, complete sequence	191	191	84%	1e-45	94%	CP003151.1
Borrelia garinii NMJW1, complete genome	185	185	84%	7e-44	94%	CP003866.1
Borrelia bissettii DN127, complete genome	185	185	84%	7e-44	94%	CP002746.1
Borrelia garinii PBi, complete genome	185	185	84%	7e-44	94%	CP000013.1

Alignments

Borrelia burgdorferi CA382, complete genome

Sequence ID: **gb|CP005925.1|** Length: 910736 Number of Matches: 1

Range 1: 398068 to 398191
