# Supplementary material for: Culture and identification of Borrelia spirochetes in human vaginal and seminal secretions
Source: F1000Res. 2015 Apr 27;3:309. Originally published 2014 Dec 18. [Version 3] doi: 10.12688/f1000research.5778.3 (PMC5482345; doi:10.12688/f1000research.5778.3)
Supplement: Updated data of Borrelia spirochetes in human vaginal and seminal secretions — The dataset contains data files 1, 2, 2a, 3, 3a, 4, 5, 5a, 6 and 7. Detailed legends for each files can be found in the text file provided. [file f1000research-3-6856-s0000.tgz › 3a95801b-ea1b-4731-9d1e-5376b3b8a9eb_Data_new.zip!/Data 7/PCRBlood UNH.pdf]

Fla Sequence

Patient 2 blood culture
>P2bc_fla_F_PREMIX Sample_Name=P2bc_fla_F_PREMIX Chromat_id=2243002 Read_id=2219379 Version=1 Length=602
GAAATTAATAGAATTGCTGATCAAGCTCAATATAACCAAATGCACATGTTAT
CAAACAAATCTGCTTCTCAAAATGTAAGAACAGCTGAAGAGCTTGGAATGCA
GCCTGCAAAAATTAACACACCAGCATCACTTTCAGGGTCTCAAGCGTCTTGG
ACTTTAAGAGTTCATGTTGGAGCAAACCAAGATGAAGCTATTGCTGTAAATA
TTTATGCAGCTAATGTTGCAAATCTTTTCTCTGGTGAGGGAGCTCAAACTGCT
CAGGCTGCACCGGTTCAAGAGGGTGTTCAACAGGAAGGAGCTCAACAGCCA
GCACCTGCTACAGCACCTTCAAATT

100% B31

Patient 3 blood culture
>P3bc_fla_F_PREMIX Sample_Name=P3bc_fla_F_PREMIX Chromat_id=2242973 Read_id=2219386 Version=1 Length=657
TTACAGACGAAATTAATAGAATTGCTGATCAAGCTCAATATAACCAAATGCA
CATGTTATCAAACAAATCTGCTTCTCAAAATGTAAGAACAGCTGAAGAGCTT
GGAATGCAGCCTGCAAAAATTAACACACCAGCATCACTTTCAGGGTCTCAAG
CGTCTTGGACTTTAAGAGTTCATGTTGGAGCAAACCAAGATGAAGCTATTGCT
GTAAATATTTATGCAGCTAATGTTGCAAATCTTTTCTCTGGTGAGGGAGCTCA
AACTGCTCAGGCTGCACCGGTTCAAGAGGGTGTTCAACAGGAAGGAGCTCAA
CAGCCAGCACCTGCT

100% B31

Patient 4 blood culture
>P4bc_fla_F_PREMIX Sample_Name=P4bc_fla_F_PREMIX Chromat_id=2242967 Read_id=2219322 Version=1 Length=643
TTAATAGAATTGCTGATCAAGCTCAATATAACCAAATGCACATGTTATCAAA
CAAATCTGCTTCTCAAAATGTAAGAACAGCTGAAGAGCTTGGAATGCAGCCT
GCAAAAATTAACACACCAGCATCACTTTCAGGGTCTCAAGCGTCTTGGACTTT
AAGAGTTCATGTTGGAGCAAACCAAGATGAAGCTATTGCTGTAAATATTTAT
GCAGCTAATGTTGCAAATCTTTTCTCTGGTGAGGGAGCTCAAACTGCTCAGGC
TGCACCGGTTCAAGAGGGTGTTCAACAGGAAGGAGCTCAACAGCCAGCACCT
GCTACAGCACCTTCAAACNAGAGAAAAGATTTGCAACATTAACTGCNTAAAT
ATTTNCAGCAATAGCTTCATCATGGTTTGCTCCAACTTGAACTCTTAAAGTCA
ACACGCTGANACCNTGAGGTGATG

99% B31

16S rRNA Sequence

Patient 6 blood culture
>P6bc_16S_PREMIX Sample_Name=P6bc_16S_PREMIX Chromat_id=1482621
Read_id=1462877 Version=1 Length=415
TGTAGCATACATTCAGTGGCGAACGGGTGAGTAACGCGTGGATGATCTACCT
ATGAGATGGGGATAACTATTAGAAATAGTAGCTAATACCGAATAAGGTCAGT
TAATTTGTTAATTGATGAAAGGAAGCCTTTAAAGCTTCGCTTGTAGATGAGTC
TGCGTCTTATTAGCTAGTTGGTAGGGTAAATGCCTACCAAGGCAATGATAAG
TAACCGGCCTGAGAGGGTGAACGGTCACACTGGAACTGAGATACGGTCCAGA
CTCCTACGGGAGGCAGCAGCTAAGAATCTTCCGCAATGGGCGAAAGCCTGAC
GGAGCGACACTGCGTGAATGAAGAAGGTCGAAAGATTGTAAAATTCTTTTAT
AAATGAGGAATAAGCTTTGTAGGA

Sequence has one nucleotide insertion 389/390. 99% identity to B31
