# Supplementary material for: Culture and identification of Borrelia spirochetes in human vaginal and seminal secretions
Source: F1000Res. 2015 Apr 27;3:309. Originally published 2014 Dec 18. [Version 3] doi: 10.12688/f1000research.5778.3 (PMC5482345; doi:10.12688/f1000research.5778.3)
Supplement: Updated data of Borrelia spirochetes in human vaginal and seminal secretions — The dataset contains data files 1, 2, 2a, 3, 3a, 4, 5, 5a, 6 and 7. Detailed legends for each files can be found in the text file provided. [file f1000research-3-6856-s0000.tgz › 3a95801b-ea1b-4731-9d1e-5376b3b8a9eb_Data_new.zip!/Data 7/PCRSemVag UNH.pdf]

16S rRNA

Patient 3 Seminal Culture

>Bb16S_P3sem_F_PREMIX Sample_Name=Bb16S_P3sem_F_PREMIX
Chromat_id=2059380 Read_id=2036284 Version=1 Length=238
AGCTTTGTAGGACCTGGCTTAGAACTAACGCTGGCAGTGCGTCTTAAGCATG
AGGAATAAGCTTTGTAGGACCTGGCTTAGAACTAACGCTGGCAGTGCGTCTT
AAGCATGAGGAATAAGCTTTGTAGGACCTGGCTTAGAACTAACGCTGGCAGT
GCGTCTTAAGCATGAGGAATAAGCTTTGTAGGACCTGGCTTAGAACTAACGC
TGGCAGTGCGTCT

99% B31 sequence

PyrG

Patient 12 Seminal Culture

>P12sem_PYRG_PREMIX Sample_Name=P12sem_PYRG_PREMIX
Chromat_id=3150347 >Read_id=3112242 Version=1 Length=692
NNNNNNNNNNNNNCTNNNAGNNTANTTAATAGCCATAATTTTACCTTCATAT
CCTTTGCCTCCAAAGCCGCCAGGAACAATAATGCCGTCAAACTCTTTTAAACA
GCTCTCATTTAAATCATTAGAATCAATTAAAGTGCTTTTAATAAGCAAATCCA
AATGGGCTGCAACATGAACCAAAGACTCTCTAATTGATGCATAAGAATCATC
AAGTTCAGCATATTTACCACAAATAGCAATATTAATAATTTTTTTAGGCACAA
AAAAATTAGATTTTATAACTCCTACAAGCTTTGAAAGCTCTTCTATTTTTGGA
TCAACCTTAATATTTAACTTAGAGCTTAAAATCTCATGTACACCCTGCTTATA
AAAAGATATAGGAATTTCATAAATAGTAGAAACATCAACATTGTCAATAATA
GAAGTGCTCTCAACATTGCAAAACATTGCCACTTTTTTTCTGATTTGGTCTGTC
AATACTTGTGAACTTCTAGCAATAATTAAATCGGGGAAAATACCTGCTTTATT
TAAGGTTTTAACACTTTGTTGAGTAGGTTTAGATTTTTGCTCATTAATTCCAGC
TGGACTTGGCACATATGTTAAATGAATAAAAGAAATATTACCACTCCCAATC
TCCTGTCTTATTTGTCTTACTGTCTCAATAAATAAAATATTTTCCATATCAANN
NNNN

99% B31 sequence
