# Supplementary material for: Culture and identification of Borrelia spirochetes in human vaginal and seminal secretions
Source: F1000Res. 2015 Apr 27;3:309. Originally published 2014 Dec 18. [Version 3] doi: 10.12688/f1000research.5778.3 (PMC5482345; doi:10.12688/f1000research.5778.3)
Supplement: Peer review history

# Peer review: Referee response for version 3

**DOI:** 10.5256/f1000research.6856.r23250

**Published:** 2017-06-16

**Authors:** Nataliia Rudenko, Maryna Golovchenko

**Affiliations:** 1 Institute of Parasitology, Biology Centre Czech Academy of Sciences, České Budějovice, Czech Republic

The third version of the paper from Middelveen *et al.*, "Culture and identification of *Borrelia *spirochetes in human vaginal and seminal secretions" represents a well designed and nicely executed study strongly supported by multiple results that were obtained by three independent laboratories. The impact of the presented data is significant as it highlights the alternative way of the distribution of the causative agent of Lyme disease, *Borrelia burgdorferi sensu stricto *spirochetes, rather than the traditionally accepted vector-borne way. The well balanced study presents the cultivation of live and replicating spirochetes from genital secretions, confirming the possibility of person-to-person transmission of Lyme disease spirochetes. Supported by multiple techniques, the obtained results are clear, consistent and confirming. The authors do not bring the conclusion or speculate that spirochetes transmitted from person-to-person might trigger Lyme disease in humans, as this postulation needs to be deeply studied. However, confirmation of the fact that live Borrelia are present in semen and vaginal secretions of patients with Lyme disease and can be cultured in active and replicating form from them is the first and significant step in analysis of person-to-person transmission of the causative agent of Lyme disease. This may be one of the reasonable explanations of the increased numbers of confirmed cases of Lyme disease worldwide, in addition to the others that are not discovered yet.

We have read this submission. We believe that we have an appropriate level of expertise to confirm that it is of an acceptable scientific standard.

# Peer review: Referee response for version 3

**DOI:** 10.5256/f1000research.6856.r8477

**Published:** 2015-05-11

**Authors:** Monica E. Embers

**Affiliations:** 1 Division of Bacteriology and Parasitology, Tulane National Primate Research Center, Tulane University Health Sciences, Covington, LA, USA

My concerns have not necessarily been allayed by the authors' rebuttal.

Please be aware that I have invested as much time as I can in the review process for this article, and will be unable to contribute further.

I have read this submission. I believe that I have an appropriate level of expertise to state that I do not consider it to be of an acceptable scientific standard, for reasons outlined above.

# Peer review: Referee response for version 3

**DOI:** 10.5256/f1000research.6856.r8478

**Published:** 2015-04-28

**Authors:** Robert Smith

**Affiliations:** 1 School of Life Sciences, University of Glasgow, Glasgow, UK

This current version of the paper is one which contributes significantly to the field. I re-affirm my original approval of what I consider is a well executed and well presented study and is worthy of indexation for rational scrutiny and further investigations by the wider medical and scientific communities.

I have read this submission. I believe that I have an appropriate level of expertise to confirm that it is of an acceptable scientific standard.

# Peer review: Referee response for version 2

**DOI:** 10.5256/f1000research.6473.r8176

**Published:** 2015-04-09

**Authors:** Monica E. Embers

**Affiliations:** 1 Division of Bacteriology and Parasitology, Tulane National Primate Research Center, Tulane University Health Sciences, Covington, LA, USA

The design of these experiments to test the hypothesis that viable spirochetes exist in genital secretions is appropriate. However, the implementation of the experiments, presentation, and interpretation of data is questionable. 

Culture of *B. burgdorferi* from secretions: These are not sterile sources, yet there is no mention of contamination with other microbes. The authors should show (supplemental data) staining of secretions and cultures from healthy controls. One would expect some level of background staining.

Images shown in Figures 1, 2 and 3 are not specifically detecting spirochetes and are questionable without appropriate controls. The darkfield and silver stain are of very little use, given that “pseudospirochetes” of flagellar bundles can forms in body fluid cultures. No positive or negative controls are shown. The straight morphology and varying lengths of that depicted in Figure 1A (and in all of the dataset 1 darkfield samples provided) are inconsistent with *B. burgdorferi* morphology. Specific staining shown in Fig 2A is more consistent with spirochete morphology and a legitimate detection method (albeit a low quality image). However, the authors indicate in the Methods section that DAPI counterstain was used but no blue staining is apparent. Does this mean that there were no other sources of DNA in the culture (bacteria or cells)? Clearly, there is ample contamination with other bacteria (seen in darkfield images of dataset 1). Concerning immunostaining with the FlaB probe, the methods are not described and no positive or negative control samples are shown. With the exception of Patient 10, none of the silver stained samples resemble spirochetes. 

This reviewer encourages readers to examine the images provided in supplementary datasets.

Concerning the molecular detection, the sequence for the 23mer FlaB DNA probe should be provided, as should evidence of validation with controls. If multiple genes were amplified from cultures, then those sequences should also be provided. Assuming there was a positive control, 98-100% homology cannot distinguish between a legitimate infection and PCR contamination (with introduction of errors from PCR).  Sequences from patients 10 and 11 as *B. hermsii* is misleading, given that they are short, disconnected regions of homology. Given that 16s was used, the entire product should line up. BLAST input was not provided for Patient 12 and the region of homology for patient 13 was only 50 bp (where for patient 1, 6 and 7 it was ~120 bp).

In conclusion, this manuscript would be made much clearer if all of the control (positive and negative) data were also included. Validation of the assays employed is unapparent. The paper cited from the Australian Biologics assay indicated PCR detection of *Borrelia *in patients at a much higher frequency than IgG or IgM immunoblot detection of exposure. This is inconsistent with multiple reports. Furthermore, the PCR primers are listed as “proprietary” so an assessment of specificity cannot be made. Sending blinded samples to an impartial 3rd party for testing using validated assays is recommended.

I have read this submission. I believe that I have an appropriate level of expertise to state that I do not consider it to be of an acceptable scientific standard, for reasons outlined above.

# Response

**Published:** 2015-04-14

**Authors:** Raphael Stricker

**Affiliations:** USMA, USA

- *The design of these experiments to test the hypothesis that viable spirochetes exist in genital secretions is appropriate. However, the implementation of the experiments, presentation, and interpretation of data is questionable. *
*Culture of B. burgdorferi from secretions: These are not sterile sources, yet there is no mention of contamination with other microbes. The authors should show (supplemental data) staining of secretions and cultures from healthy controls. One would expect some level of background staining.*
  We acknowledge that these are not sterile sources. Three antibiotics were used to limit the growth of normal flora. Although *Borrelia* cultures may contain other flora, obtaining pure isolates was not the purpose of the study. The negative controls from healthy subjects did not show PCR evidence of spirochetal infection, as seen in Table 5 and Dataset 7. Real-time PCR failed to show spirochetal infection in genital cultures from negative controls (Table 5A), and both TaqMan PCR and nested PCR using a variety of targets failed to show *Borrelia* infection in genital cultures from negative controls (Table 5B). To address the referee’s concern about control silver staining and immunostaining, however, we have performed additional control silver stains and immunostains using a culture from uninfected mouse plasma, which showed no staining for *Borrelia* with either technique. Cultures of fungal-infected human skin, mixed gram positive strep and staph (from dairy mastitis cultures) and mixed gram negative fecal coliforms (from canine feces cultured on McConkey Agar) also failed to show significant immunostaining for *Borrelia*. In contrast, plasma culture from a mouse infected with *Borrelia* spirochetes processed at McClain Laboratory showed positive silver staining and immunostaining. We have included this information in Datasets 2A and 3A and added a description in the Results section on Pages 8 and 9.
- *Images shown in Figures 1, 2 and 3 are not specifically detecting spirochetes and are questionable without appropriate controls. The darkfield and silver stain are of very little use, given that “pseudospirochetes” of flagellar bundles can forms in body fluid cultures. No positive or negative controls are shown.*
  Figure 1 shows darkfield imaging and silver staining indicating that cultures are living and viable. The Figure shows typical spirochetal morphology, and these viable spirochetes were confirmed to be *Borrelia* by PCR, as shown in Table 5 and Dataset 7. Figure 2 shows immunostaining that is specific for Borrelia and correlates with positive PCR detection in these samples. Figure 3 shows molecular hybridization with the FlaB probe that is highly specific for *Borrelia*. The positive and negative controls for this experiment are shown in Supplemental Figure 1. Although each figure by itself might not indicate the presence of *Borrelia*, the combination of darkfield imaging, silver staining, immunostaining, PCR testing using a range of targets and molecular hybridization provides strong evidence for viable *Borrelia* in these genital cultures, as stated repeatedly in the article.
- *The straight morphology and varying lengths of that depicted in Figure 1A (and in all of the dataset 1 darkfield samples provided) are inconsistent with B. burgdorferi morphology.*
  We respectfully disagree with the referee, and we have included a discussion of morphological variation and pleomorphism in *Borrelia* cultures in the Discussion section on Page 16 with multiple references. Furthermore, to our knowledge nobody has ever studied *Borrelia* in seminal or vaginal cultures, so nobody knows what morphological variants would be present. Since *Borrelia* morphological variation and pleomorphism is acknowledged in the medical literature, visual identification alone is not reliable and that is why we used silver staining, immunostaining and molecular detection to characterize the isolates as *Borrelia*.
- *Specific staining shown in Fig 2A is more consistent with spirochete morphology and a legitimate detection method (albeit a low quality image). However, the authors indicate in the Methods section that DAPI counterstain was used but no blue staining is apparent. Does this mean that there were no other sources of DNA in the culture (bacteria or cells)? Clearly, there is ample contamination with other bacteria (seen in darkfield images of dataset 1).*
  We agree with the referee that immunostaining in Figure 2A is consistent with spirochete morphology. The DAPI counterstain was only used in the FlaB probe validation study, as shown in Supplemental Figure 1. We have modified the Methods description to reflect this fact.
- *Concerning immunostaining with the FlaB probe, the methods are not described and no positive or negative control samples are shown. Wth the exception of Patient 10, none of the silver stained samples resemble spirochetes. *
  The FlaB probe is a DNA probe, not an immunostain. This DNA hybridization technique is described in detail in Methods section 5 (Page 4) with appropriate references, and positive and negative controls for the technique are shown in Supplemental Figure 1. Again, we have included references to studies showing morphological variation and pleomorphism in *Borrelia* cultures in the Discussion section on Page 16.  As there are problems with specificity of visual identification using silver staining, we used this method to provide corroborative evidence only. It is the collective evidence -- including darkfield imaging, immunostaining and molecular detection of borrelial DNA that is important. We used a broad approach with various methods so that there was no reliance on any single methodology, as stated repeatedly in the article.
- *This reviewer encourages readers to examine the images provided in supplementary datasets.*
  We encourage readers to do the same. The Dataset images support the findings described in the article.
- *Concerning the molecular detection, the sequence for the 23mer FlaB DNA probe should be provided, as should evidence of validation with controls.*
  We have provided the FlaB probe sequence in Methods section 5 (Page 4), courtesy of Dr. Alan MacDonald. Again, this is a molecular technique intended to provide corroborative evidence -- along with all the other techniques. A detailed vaildation study was provided in Supplemental Figure 1 clearly showing that the molecular probe was specific for *Bb sensu stricto*.
- *If multiple genes were amplified from cultures, then those sequences should also be provided. Assuming there was a positive control, 98-100% homology cannot distinguish between a legitimate infection and PCR contamination (with introduction of errors from PCR). *
  The gene sequences are described in Table 6, and BLAST analyses are shown in Dataset 6. The argument against PCR contamination is presented in the Discussion section on Page 17.
- *Sequences from patients 10 and 11 as B. hermsii is misleading, given that they are short, disconnected regions of homology. Given that 16s was used, the entire product should line up. BLAST input was not provided for Patient 12 and the region of homology for patient 13 was only 50 bp (where for patient 1, 6 and 7 it was ~120 bp).*
  The *B. hermsii *sequences are short, but they were the closest match in those samples. The referee ignores the fact that this couple was the only one with a distinct *Borrelia* sequence that was detected in both sexual partners and not in any other individual or couple in our small study. The BLAST analysis for Patient 12 was performed using a different program, and labeling for the sequence input has been provided. For Patient 13, the region of homology was short, but it was virtually identical to the sequence in her sexual partner (Patient 12), as shown in Table 6.
- *In conclusion, this manuscript would be made much clearer if all of the control (positive and negative) data were also included. Validation of the assays employed is unapparent. The paper cited from the Australian Biologics assay indicated PCR detection of Borrelia in patients at a much higher frequency than IgG or IgM immunoblot detection of exposure. This is inconsistent with multiple reports. Furthermore, the PCR primers are listed as “proprietary” so an assessment of specificity cannot be made. Sending blinded samples to an impartial 3rd party for testing using validated assays is recommended.*
  As outlined above, we have included control data for the PCR and molecular hybridization testing, which is the “acid test” for the presence of *Borrelia* in genital cultures. The assays described in our study are internally consistent since the darkfield imaging, silver staining and immunostaining have been validated by the PCR and molecular hybridization procedures. In contrast to the referee’s comment, there is ample evidence for “seronegative” Lyme disease, and we have included a discussion of this entity on Page 16 with appropriate references. We used two independent laboratories for PCR testing and molecular hybridization studies, and the results using a range of primers are consistent between the two laboratories. We intend to send blinded samples to independent laboratories for confirmation of our results. In light of our detailed responses to the referee comments, we believe that this preliminary study merits reconsideration for approval by the referee.

# Peer review: Referee response for version 2

**DOI:** 10.5256/f1000research.6473.r7696

**Published:** 2015-03-09

**Authors:** Robert Smith

**Affiliations:** 1 School of Life Sciences, University of Glasgow, Glasgow, UK

Middelveen *et al.* have, produced a well written and informative account of their findings on the “Culture and identification of Borrelia spirochetes in human vaginal and seminal secretions” from subjects diagnosed as Lyme Disease patients by serological and clinical parameters compared to control individuals who were asymptomatic and serologically negative for the Borrelia burgodorfei. Of most interest is their report of the PCR sequencing of cultured spirochetes from three couples having unprotected sex which indicated that two couples had identical strains of *Bb sensu stricto *in their semen and vaginal secretions, while the third couple had identical strains of *B. hermsii*.

I believe the study is worthy of acceptance and approve it for the following reasons:

1. The rationale and hypothesis behind the investigation is clearly stated.
2. The work has been carefully planned, employing an array of pertinent experimental approaches: routine light microscopy, histological silver staining and immunohistological staining methodology, DNA probe analysis and PCR to demonstrate the presence of *Borrelia* spirochetes in cultures prepared from semen or vaginal secretions from Lyme disease subjects serologically tested for Bb, whilst samples from Bb negative control subjects did not yield spirochetes.
3. The study is well executed, with samples analysed following blind coding, and techniques performed in a number of independent laboratories.
4. The data sets, tables and figures are presented in a detailed and informative manner.
5. The authors demonstrate an authoritative awareness of previous work in the area, citing relevant animal studies which had investigated the possible sexual transmission of Borrelia, and give a plausible explanation for the discrepancies between the findings by Guftafson in dogs and those of Moody and Woodrun in inbred rodent models. 
6. In this second version of the article, the authors have taken pains to address the main concerns of the reviewer Donta, and also answered the comments raised by Baker and others, to version one of the manuscript.
7. In their replies, Middelveen and Stricker have, in my opinion, more than allayed the criticisms with regard to technical details, and also those concerning aspects such as CDC surveillance criteria and of the unknown spirochetal load.
8. The conclusions drawn, that the culture of viable *Borrelia *spirochetes in human genital secretions suggests that Lyme disease could be transmitted by intimate contact from person to person, are justified and of significance. Their findings to date warrant a larger study and further attention by the scientific and medical community.

I have read this submission. I believe that I have an appropriate level of expertise to confirm that it is of an acceptable scientific standard.

# Peer review: Referee response for version 1

**DOI:** 10.5256/f1000research.6178.r7090

**Published:** 2015-01-05

**Authors:** Sam T. Donta

**Affiliations:** 1 Falmouth Hospital, Falmouth, MA, USA

There are a number of issues that mitigate against the authors' conclusion that Lyme disease can be transmitted sexually. 

While there are conflicting reports from animal studies that there can be transmission by contact between animals and other studies that appear to better controlled that do not provide such evidence, there is no obvious epidemiological evidence in humans that this is a likely possibility. Apart from the sociologic implications of claiming that intimate or even casual transmission is possible, there needs to be more compelling evidence that this might be the case than is offered in this report.

Specific issues in this report:

1. No evidence that samples were blinded.
2. The numbers of patients were too small from which to draw meaningful conclusions.
3. The actual serologic data on patients should be presented in order to be more properly assessed.
4. PCR-DNA analyses should have been done on the original specimens; again in a blinded fashion, and weekly during the culture period. And, as PCR-DNA testing is much more sensitive than cultures, without this information, the validity of the presented information remains in question.
5. Concurrent samples of other body fluids, i.e. blood, saliva, should have been included.
6. Even if presuming the presence of the Lyme *borrelia *in vaginal secretions or semen, the numbers present would have not been sufficient to cause any transmission of infection, as, with any infectious process, there needs to be a critical inoculum to establish infection. This is the case with transmission by ticks.
7. *Borrelia *may be spirochetes as are *Treponemes *and *Leptospira*, but the transmission of the latter are obviously through body fluids, and not by tick or other vectors. It is not clear that infections transmitted by ticks are also transmitted by intimate or casual contact. 
8. If it was true that 8/11 samples were positive, one would expect a much more obvious clinical picture of transmission by intimate or casual contact, which is not the case.
9. PCR-DNA analyses and long-term cultures can be subject to contamination, making the data here more difficult to interpret.

I have read this submission. I believe that I have an appropriate level of expertise to state that I do not consider it to be of an acceptable scientific standard, for reasons outlined above.

# Response

**Published:** 2015-01-12

**Authors:** Raphael Stricker

**Affiliations:** USMA, USA

Co-written with Marianne J. Middelveen

- *There are a number of issues that mitigate against the authors' conclusion that Lyme disease can be transmitted sexually. *
  We did not conclude that *“Lyme disease can be transmitted sexually”*. Based on preliminary editorial comments, we were careful to state that our microscopy, immunochemistry, molecular hybridization and PCR analysis showing live, culturable *Borrelia *in semen and vaginal secretions suggests that these spirochetes could be transmitted in that manner. Our study does not prove this form of transmission, and we do not make this claim anywhere in the text.
- *While there are conflicting reports from animal studies that there can be transmission by contact between animals and other studies that appear to better controlled that do not provide such evidence, there is no obvious epidemiological evidence in humans that this is a likely possibility. Apart from the sociologic implications of claiming that intimate or even casual transmission is possible, there needs to be more compelling evidence that this might be the case than is offered in this report.*
  The statement that “better controlled” studies do not provide support for contact transmission of *Borrelia *in animals is contrary to the examples in mice and dogs described in the Introduction and Discussion sections of our article. We have pointed out that the two studies on highly inbred rodents that allegedly showed lack of such transmission did not use PCR techniques and therefore may have missed this transmission. The fact that the CDC now admits to more than 300,000 new cases of Lyme disease each year in the USA (and perhaps as many as one million new cases, as outlined in Stricker & Johnson, 2014) is suggestive that other forms of transmission occur, as noted in the Discussion section on page 10.
-  
*1. No evidence that samples were blinded. *
  In response to the referee’s comment, we have noted that the laboratory testing was performed on coded samples in a blinded fashion. This has been noted in the Abstract and reiterated throughout the Methods section.
- *2. The numbers of patients were too small from which to draw meaningful conclusions.*
  The “ *meaningful conclusions*” are that  microscopy, immunochemistry, molecular hybridization and PCR analysis demonstrates live, culturable *Borrelia *in semen and vaginal secretions from Lyme disease patients. Although the numbers are relatively small, we feel that our detailed study supports this conclusion.
- *3.The actual serologic data on patients should be presented in order to be more properly assessed.*
  We can include the actual serologic data as an original Dataset, but we don’t see how that would alter the experimental findings in our study. The serologic data is presented in Table 1 and the Results section. In response to the referee’s comment, we have added more detail about the serologic and diagnostic criteria in the Methods section on page 4, with supporting references. 
- *4. PCR-DNA analyses should have been done on the original specimens; again in a blinded fashion, and weekly during the culture period. And, as PCR-DNA testing is much more sensitive than cultures, without this information, the validity of the presented information remains in question.*
  PCR-DNA analysis was done on the “ *original specimens”* in a blinded fashion  in conjunction with the microscopy, immunochemistry and molecular hybridization analysis. Repeated testing at weekly or other intervals is beyond the scope of this pilot study, and this type of testing should certainly be explored in future Lyme disease studies.
- *5. Concurrent samples of other body fluids, i.e. blood, saliva, should have been included.*
  As shown in Table 5, we did do PCR testing on concurrent blood samples in some patients. Saliva testing for *Borrelia *would have been of interest, but this form of *Borrelia *testing requires further investigation and is beyond the scope of our study.  
- *6. Even if presuming the presence of the Lyme borrelia in vaginal secretions or semen, the numbers present would have not been sufficient to cause any transmission of infection, as, with any infectious process, there needs to be a critical inoculum to establish infection. This is the case with transmission by ticks.*
  In response to the referee’s comment, we have researched the number of spirochetes necessary for transmission of *B. burgdorferi* infection in mice and *T. pallidum * infection in humans. The results show that a very small number of spirochetes (as little as 18 organisms) are required for transmission of infection, and we have included this information in the Discussion on page 11. We have also noted that seminal plasma and the female genital tract may provide a relatively permissive environment for spirochetes compared to blood, skin and other immune sites, making transmission theoretically easier via the genital route.
- *Borrelia may be spirochetes as are Treponemes and Leptospira, but the transmission of the latter are obviously through body fluids, and not by tick or other vectors. It is not clear that infections transmitted by ticks are also transmitted by intimate or casual contact.*
  In response to the referee’s query, we have provided examples of other agents ( *Babesia*, *Chlamydia*, *Coxiella*) that are proven or postulated to be transmitted by both tickbite and intimate contact. This information is included in the Discussion on page 11 with supporting references.
- *If it was true that 8/11 samples were positive, one would expect a much more obvious clinical picture of transmission by intimate or casual contact, which is not the case.*
  As noted above, the substantial numbers of  new Lyme disease cases each year suggests additional forms of transmission beyond a tickbite. At this time, the true epidemiology of Lyme disease is unknown because the CDC surveillance system only captures less than 10% of Lyme disease cases, as noted in the Discussion on page 11. Other epidemiological studies have suggested that some infected patients may be relatively asymptomatic ( Harvey & Salvato, 2003), so transmission via intimate contact resulting in less obvious infection is plausible. The risk of this form of transmission and correlation with symptoms merits further study. Our report simply raises the possibility, and rejecting the report will shove this issue under the rug to the detriment of Lyme patients.
- *9. PCR-DNA analyses and long-term cultures can be subject to contamination, making the data here more difficult to interpret.*
  PCR-DNA analysis is subject to contamination. That is why we did blinded testing that always included negative controls in three different laboratories using microscopy, immunochemistry and molecular hybridization to confirm the PCR findings. Although PCR testing alone might be “difficult to interpret”, the combination of experimental techniques done in different laboratories makes interpretation much more reliable.

We hope that the referee will change his opinion after reading the revised manuscript and our responses to his comments.
